# Valorization of natural adsorbents for removing chromium (VI) from industrial wastewater: a review

**DOI:** 10.3389/fchem.2025.1608863

**Published:** 2025-06-12

**Authors:** Hajira Haroon, Tayyab Ashfaq Butt, Jehanzeb Ali Shah, Alin Ciobica, Laura Ecaterina Romila, Vasile Burlui, Hamida Bibi, Muhammad Bilal

**Affiliations:** ^1^ Department of Environmental Sciences, University of Haripur, Haripur, Pakistan; ^2^ Department of Environmental Sciences, COMSATS University Islamabad, Abbottabad, Pakistan; ^3^ Department of Civil Engineering, College of Engineering, University of Hail, Ha’il, Saudi Arabia; ^4^ “Ioan Haulica” Institute, Apollonia University, Iasi, Romania; ^5^ Department of Biology, Faculty of Biology, “Alexandru Ioan Cuza” University of Iasi, Iasi, Romania; ^6^ CENEMED Platform for Interdisciplinary Research, University of Medicine and Pharmacy “Grigore T. Pop”, Iasi, Romania; ^7^ “Olga Necrasov” Center, Depart. of Biomedical Research, Romanian Academy, Iasi, Romania; ^8^ Academy of Romanian Scientists, Bucuresti, Romania; ^9^ Department of Environmental Science, Abdul Wali Khan University Mardan, Mardan, Pakistan

**Keywords:** adsorption, adsorbents, activation, batch experiments, chromium (VI)

## Abstract

Chromium (Cr(VI)) is often released from various industries in excess in developing countries, which constitutes non-compliance with environmental regulations. This metal is hazardous for the aquatic ecosystem and is responsible for toxicity, carcinogenicity, and mutagenicity in humans. Adsorption is an effective and relatively inexpensive approach for treating the excess Cr(VI) compared to conventional methods. Commercially available adsorbents cannot be considered economical yet for industrial applications, which has alternatively resulted in the use of natural adsorbents. The current study focuses on Cr(VI) removal using low-cost natural adsorbents and discusses the different conditions used for such treatments. Previous studies have shown the following order of average Cr(VI) removal using different adsorbents: leaves > bark > agriculture > dry shell > tea = fungi > yeast > algae > sawdust > bacteria. Moreover, acid modification has been reported to offer the best results. The adsorption data are best fitted to both the Langmuir and Freundlich isotherms. Hence, the abovementioned low-cost natural biomasses hold promising potential for Cr(VI) removal from wastewater, whose removal efficiency can be improved by adopting economical and effective pretreatment techniques. Literature also shows that leaves are more efficient and economical for Cr(VI) removal without pretreatment from among the various available bulk biomasses. The present review is expected to provide guidance for low-cost treatment of Cr(VI) at the industrial scale.

## 1 Introduction

The presence of toxic metals in industrial wastewater, marine water, and freshwater has become a global environmental issue (Ajmal et al., 2003; Kargar et al., 2011; Mzoughi and Chouba, 2011; Zou et al., 2019). Heavy metals poses serious threats to streams and lakes (Ghaderi et al., 2012; Alimohammad Kalhori et al., 2011; Ashraf et al., 2011; Hashem et al., 2017; Tripathi et al., 2024). Among the various heavy metals, Cr is often released into water bodies in the form of industrial wastewater. Over the past few decades, large quantities of wastes containing Cr have been directly discharged into the environment without any treatments (Qiang et al., 2011). The toxicity of Cr varies with its oxidation state. In natural water, Cr usually exists in two main oxidation states, namely, trivalent chromium (Cr(III)) and hexavalent chromium (Cr(VI)) (Sharma et al., 2022). Compared to Cr(III), Cr(VI) is 100–1000 times more toxic to organisms (Roşca et al., 2023; Chanda et al., 2024) because it has strong oxidizing ability and mobility; it is absorbed through the skin and also readily transported into the soil (Karthikeyan et al., 2005; Demirbas et al., 2008).

**FIGURE 1:**
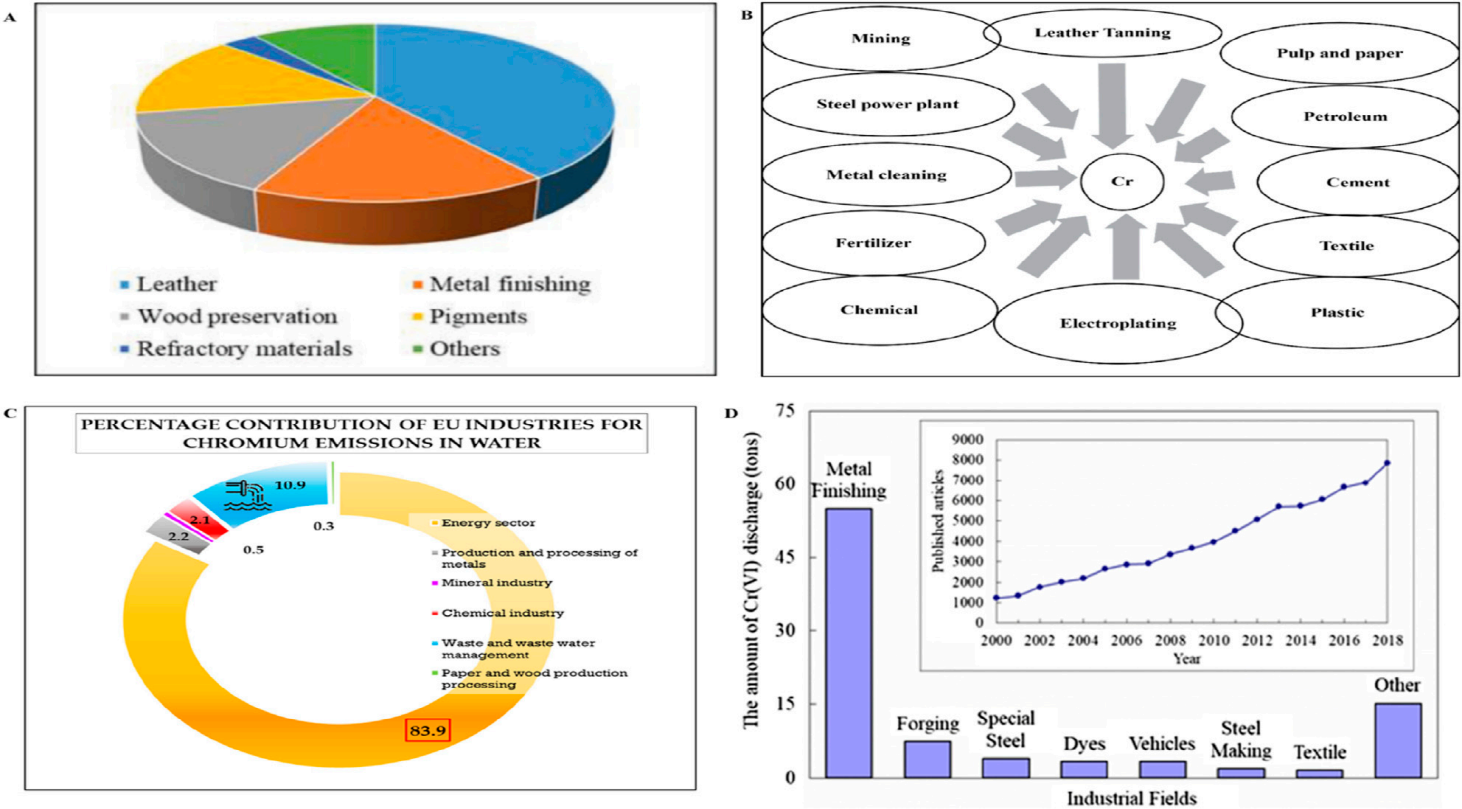
**(A)** Cr(VI) consumption in various industries (Kumar et al., 2024); **(B)** different industrial sources contributing to Cr(VI) pollution in water; **(C)** percentage contributions of EU industries to Cr in water (Tumolo et al., 2020); **(D)** Cr(VI) discharge from various industries (Xia et al., 2020).

Cr(VI) is included in the priority list of hazardous substances of the Comprehensive Environmental Response Compensation and Liability Act (CERCLA) (Moussavi and Barikbin, 2010; Peng et al., 2019). Cr(VI) is used in various industries, and the major industrial sources of Cr(VI) in wastewater are tanning, metal processing, electroplating, wood preservative, pigment, paint, steel fabrication, textile, dyeing, and canning industries, as shown in Figure 1 (Demirbas et al., 2008; Moussavi and Barikbin, 2010; Singh et al., 2022). According to the US Environmental Protection Agency, the permissible limits for Cr(VI) are 0.1 and 0.05 mg L^−1^ in inland surface water and drinking water, respectively (Qiang et al., 2011; Brusseau and Artiola, 2019).

**FIGURE 2:**
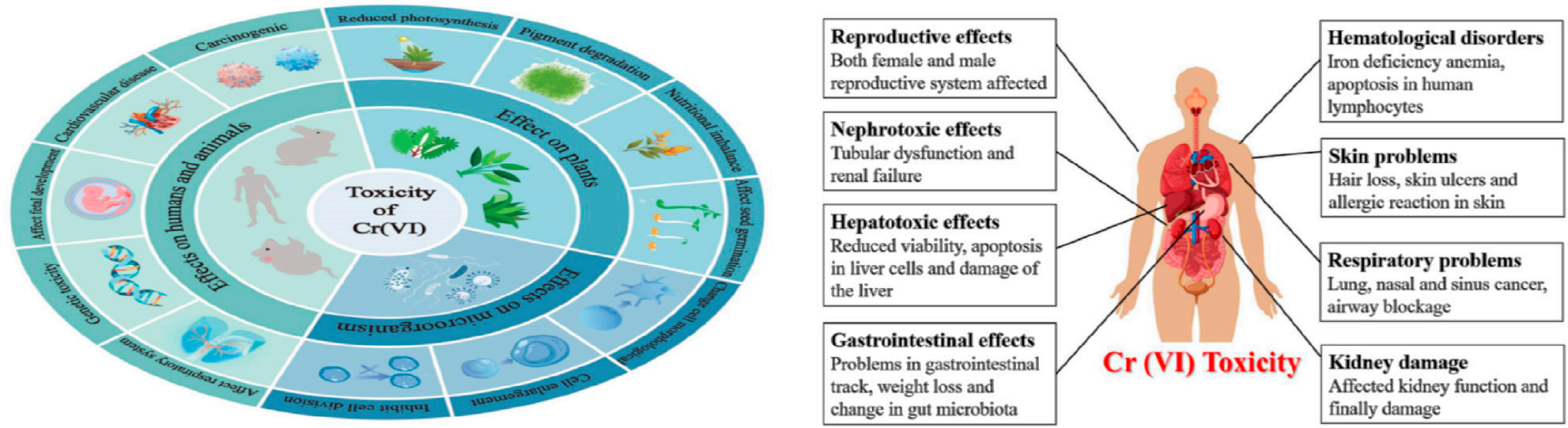
Toxicity of Cr(VI) on various living organisms (Zeng et al., 2025).

It has been shown that excess intake of Cr by humans results in many diseases (Figure 2), such as damage of the digestive tract, lung cancer, gastrointestinal issues, central nervous system irritation, capillary damage, and hepatic and renal damage (Martins et al., 2006; Kiran et al., 2007; Wan Ngah and Hanafiah, 2008; Boyle et al., 2025). Different methods are available for the treatment of Cr(VI), such as ion exchange (Wang and Lin 2008; Yuan et al., 2008; Kim et al., 2008; Li et al., 2017), ultrafiltration (Muthumareeswaran et al., 2017), nanofiltration, microfiltration (Chen et al., 2010; Zolfaghari and Kargar, 2019), chemical precipitation (Peng et al., 2018), electrochemical reduction (Peng et al., 2019), solvent extraction, reverse osmosis (Çimen, 2015), cementation, electrodialysis, electrocoagulation (Muhammad et al., 2006; Ahmet and Tuzen, 2008; Tarun et al., 2008; Cristina et al., 2009; Dang et al., 2009; Sibel et al., 2009; Zainul et al., 2009; Suresh et al., 2010), and adsorption (Monser and Adhoum, 2002; Erdem et al., 2004; Cherdchoo et al., 2019; Sirusbakht et al., 2018; Haroon et al., 2017). These methods are effective for concentrations of 1–100 mg L^−1^; however, they involve high operating and capital costs (Liang et al., 2009). The waste generated after chemical precipitation also pose disposal pollution risks and hazards to the environment. Hence, efficient and cost-effective alternative methods need to be developed for scale-up applications (Oilgae, 2010).

**FIGURE 3:**
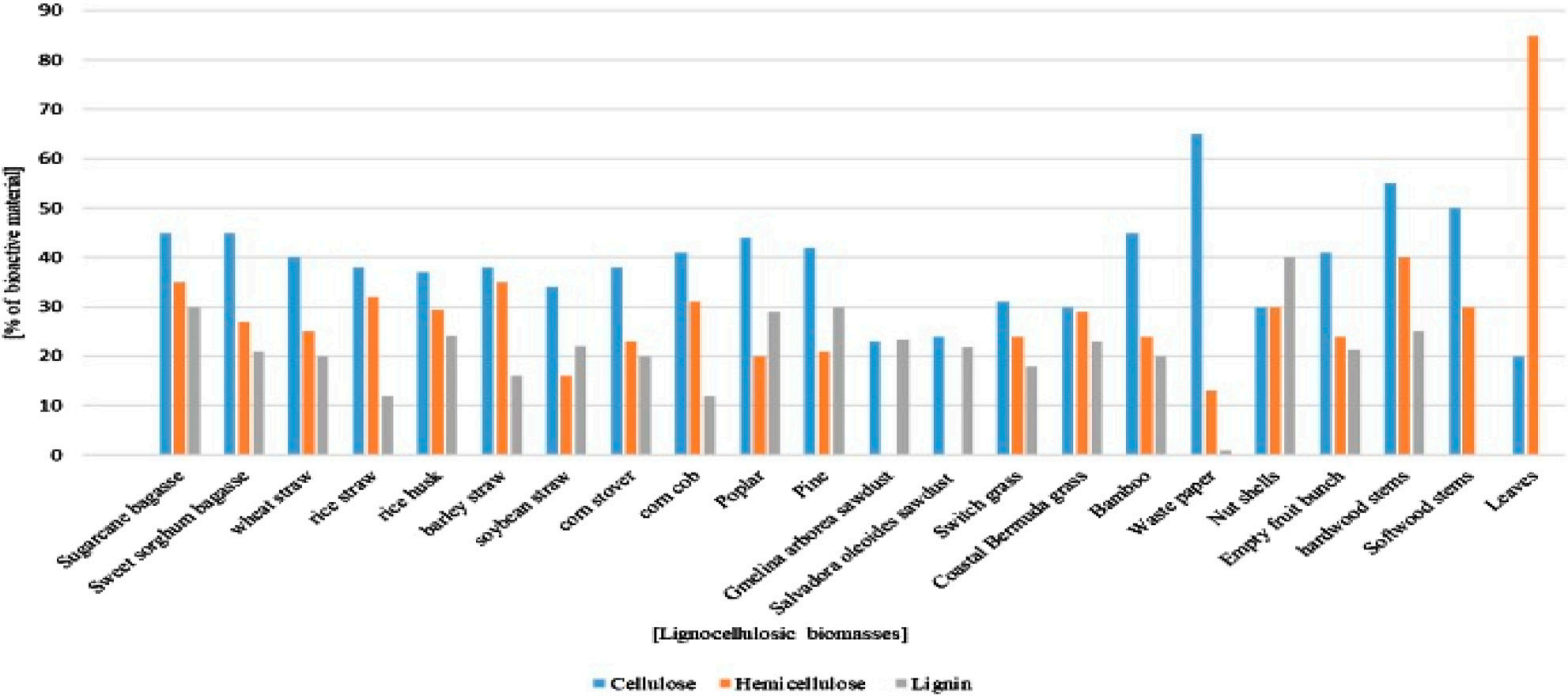
Percentage compositions of various lignocellulosic biomasses (Lee et al., 2014; Baruah et al., 2018).

Metal removal using adsorbents has distinct advantages over other conventional methods. Adsorbents are usually inexpensive, efficient (Sheraz et al., 2024), and prone to producing lower quantities of sludge after treatment (Sud et al., 2008; Satyam and Patra, 2024). Moreover, another advantage of adsorbent-based methods is that the statutory discharge limits for industrial wastewater, including enhancement of their environmental responsibility and corporate social profiles, are relatively easier to meet. Various adsorbents can be used for metal removal, such as those based on nanoparticles (Rong et al., 2024; Wenjie et al., 2022; Tesnim et al., 2024), nanocomposites (Katiyar and Katiyar, 2024), chitosan-based composites (Zhou et al., 2025), CeO_2_-based functional materials (Zhang et al., 2025), and deep-eutectic-solvent-assisted functional materials (Nie et al., 2025). Many researchers have used agriculture feedstock for the treatment of heavy metal pollutants (Odoemelam et al., 2011; Gupta et al., 2009; Subbaiah et al., 2011; Opeolu, 2009), in addition to abundantly available organic materials. Agricultural or lignocellulosic products (Figure 3) have unique chemical compositions (Lee et al., 2014; Baruah et al., 2018) and relatively high numbers of surface functional groups (i.e., phenolic and carboxylic groups) that enable successful uptake of metals from wastes and are also useful for removing Cr(VI) from wastewater (Harmon et al., 2007; Opeolu et al., 2010). Chemical binding sites comprising carboxyl (COO), carbonyl (CO), hydroxyl (OH), amine (NH_2_), or amide (RC (O) NH_2_) groups are mostly present on biomass surfaces (Barriada et al., 2009). Polar-functional-group-containing compounds are also available in agricultural byproducts, such as alcohols (R-OH), aldehydes (HCOH), ketones (RCOR), carboxylates (RCOO), phenols (C_6_H_5_-OH), and ethers (R-O-R); these bind heavy metals through hydrogen replacement or complex formation by electron pair donation (Ofomaja and Ho, 2007). Different activated and non-activated bioadsorbents, including pine wood sawdust (Sirusbakht et al., 2018), Indian jujube (Ahmad et al., 2017), coffee pulp (Gómez Aguilar et al., 2019), coffee grounds (Cherdchoo et al., 2019), almond green hulls (Nasseh et al., 2016), groundnut shells (Bayuo et al., 2019), palm kernel shells (Razavi Mehr et al., 2019), *Arachis hypogea* (peanut) leaves (Ahmad et al., 2017), green moringa tea leaves (Timbo et al., 2017), *Cassia fistula* (Amaltas) leaves (Ahmad et al., 2017), *Euclea schimperi* leaves (Gebrehawaria et al., 2015), *Acacia albida* bark (Gebrehawaria et al., 2015), *Eucalyptus camaldulensis* sawdust (Haroon et al., 2016), sugarcane bagasse (Homagaia et al., 2010), white cedar sawdust (Haroon et al., 2017), and pea seed shells (Kebede et al., 2022), have been used as economical alternatives to expensive adsorbents.

Several agricultural byproducts and natural materials have been explored for Cr(VI) adsorption, but a critical gap remains in systematically comparing their effectiveness and evaluating the impacts of chemical modifications. The present review addresses this gap by offering detailed comparisons of a wide range of unmodified and chemically modified natural adsorbents for Cr(VI) removal. As a pioneering effort, we compile and analyze the Cr(VI) removal potentials of various natural biomasses in this study, including leaves, bark, shells, and agricultural residues, as well as rank them based on performance. Moreover, this review uniquely emphasizes the roles of chemical modifications, including acids, bases, and salts, in enhancing the adsorption capacities of various natural organic (lignocellulosic) materials. In highlighting these novel features, we not only combine various sources available in literature but also propose a framework for future directions for developing cost-effective, scalable, and sustainable treatment technologies using natural organic adsorbents.

## 2 Adsorption of Cr(VI) using various waste biomasses

### 2.1 Sawdust

Aspen sawdust is a lignocellulosic solid waste that is reported to be an effective adsorbent of Cr(VI) from industrial effluents. The relationship between the adsorption capacity and temperature reflects the endothermic nature of the adsorption process. The data display a good fit for the Freundlich isotherm rather than the Temkin and Langmuir isotherms, revealing the homogeneous and monolayer adsorption process. It was observed that Cr(VI) removal increased with the adsorbent dose (3 g) because of the availability of more adsorbent (sawdust) surface. The maximum removal of Cr(VI) was noted at a pH of 2, which is attributed to the large number of positive hydrogen ions on the adsorbent surface, resulting in more electrostatic attraction between the positive adsorbent and anionic Cr. The sawdust of other types of woods used in buildings and the furniture industry is available abundantly (Mosavian et al., 2012). Neem and mango sawdust showed 80% and 60% adsorption for Cr(VI) removal, respectively, at an acidic pH of 2; this is owed to the more positive charges in the aqueous media, which makes the adsorbent surface positive and results in the attraction of more negative ions of Cr toward the sawdust adsorbent surface. Cr(VI) removal using neem sawdust revealed the best fitting to the Langmuir isotherm and followed a pseudo-second-order kinetics (Vinodhini and Das, 2010), revealing the monolayer and homogeneous surface of the adsorbent as well as chemosorption nature of the adsorption process. Studies have shown that when the free ionic sites are occupied by anionic species like HCrO_4_^−^, CrO_4_^−2^, and Cr_2_O_7_^2-^, the adsorption on the adsorbent surface decreases at pH values above 4 (Das et al., 2000; Dönmez and Aksu, 2002). This concept is further supported by the findings of Popuri (2007), who showed the competition between the oxyanion form of Cr and OH^−^, where the adsorption of Cr decreased at high pH. Politi and Sidiras (2012) observed 95.6% removal potential of Cr using pine sawdust at pH 1.2; they reported that the percentage removal increased linearly with the initial amount of Cr(VI) and decrease in pH.

In another study, neem sawdust was used for Cr(VI) treatment in a fixed bed column; here, maximum removal was observed at pH 2 due to protonation of the adsorbent surface and its electrostatic attraction to the anionic chromate ions. However, at higher pH, Cr(VI) removal decreased because of the competition between hydroxyl ions and oxyanions of Cr. The effects of co-ions on Cr(VI) removal were also studied, and it was found that cations (Na^+^ and Mg^2+^) did not affect adsorption, while anions (Cl^−^) interfered with adsorption by competing with the Cr species. The surface area of the neem sawdust was reported to be 3.76 m^2^ g^−1^; it was found that the adsorption data were well fitted to the bed depth service time (BDST) model and that the estimated Cr(VI) uptake capacity was 33,009.9 mg g^−1^; the column breakthrough time also decreased in the presence of co-ions (Vinodhini and Das, 2010).

White cedar sawdust has been reported to aid Cr(VI) removal by up to 62% under optimum conditions (Haroon et al., 2017). *Ziziphus mauritiana* (Indian jujube) sawdust also showed a monolayer adsorption capacity of 3.66 mg g^−1^ for Cr(VI) removal (Ilyas et al., 2014; Ahmad et al., 2017). Pine wood sawdust with a Brunauer–Emmett–Teller (BET) surface area of 0.434 m^2^ g^−1^ also revealed maximum Cr(VI) adsorption at acidic pH (Sirusbakht et al., 2018). *E. camaldulensis* sawdust (ECS) has been explored for Cr(VI) removal in both batch and column experiments. In the batch study, ECS showed a maximum uptake of 35.58 mg g^−1^ at pH = 2 through chemisorption, as confirmed by Cr(VI) desorption of only 15% with potassium hydroxide. The column study revealed that the experimental data best fitted the BDST model with an adsorption capacity of 2,443.636 mg L^−1^ at 60% breakthrough (Haroon et al., 2016). Local market-generated sawdust has also been shown to allow a maximum Cr(VI) removal of 96.4% (Erwa and Ibrahim, 2016). A comparison of all plant-derived biomasses used for Cr(VI) adsorption is given in Table 1.

**TABLE 1:** Comparative analysis of the process variables for the adsorption of Cr (VI) using various plant-derived adsorbents.

Plant	Part	Modified with	pH	Metal concentration (mg/L)	Time (min)	Dose (g/L)	T (^o^C)	Mixing speed (rpm)	Removal (%)	Reference
*Sawdust*										
Aspen	Sawdust	NG	2.0	0.5–5	15	3	25	300	NG	Mosavian et al. (2012)
*Prosopis glandulosa*	Sawdust	NG	4.0	110	50	0.8	50	200	65	Salman et al. (2025)
*Emblica officinalis*	Sawdust	NG	2.0	500	100	2.5	40	NG	83	Kushwaha and Chakraborty (2021)
Neem	Sawdust	NG	2.0	10–150	5–360	2	28	120	80	Vinodhini and Das (2009)
Mango	Sawdust	NG	2.0	10–150	5–360	2	28	120	60	Vinodhini and Das (2009)
Pine	Sawdust	NG	2.5	1.6–7.7	NG	4	23	600	95.6	Politi and Sidiras (2012)
Poplar and fir	Sawdust	NG	3.0	NG	60	NG	NG	NG	NG	Šćiban and Klašnja (2002)
White cedar	Sawdust	NG	2.0	10–150	180	3	35	220	62	Haroon et al. (2017)
Indian jujube	Sawdust	NG	2–3	20–60	360	1	40	NG	99.9	Ilyas et al. (2014)
Mixed plants (market)	Sawdust	NG	4.0	5–200	120	NG	30	NG	96.40	Erwa and Ibrahim (2016)
Pine wood	Sawdust	NG	3.0	NG	120	3	40	220	NG	Sirusbakht et al. (2018)
Eucalyptus	Sawdust	NG	2.0	10–150	360	0.2	50	NG	71.16	Haroon et al. (2016)
Indian jujube	Sawdust	NG	2.0	NG	120	NG	40	180	NG	Ahmad et al. (2017)
Red pine	Sawdust	NG	3.0	NG	50	1.6	25	NG	55.30	Gode et al. (2008)
Teak wood	Sawdust	HCl	3–4	10–80	60	10	NG	NG	98	Ramirez et al. (2020)
*Dalbergia sissoo*	Sawdust	Formaldehyde	2.0	0.005–0.05	30	2	NG	100	85.4	Pal and Kaur (2013)
Local factory wood	Sawdust	Diethyltriamine	3.0	5–70	120	NG	NG	180	75	Elhami and Bahadori (2015)
Red pine	Sawdust	NaOH	3.0	NG	120	Ng	25	180	87.70	Gode et al. (2008)
Red pine	Sawdust	Tartaric acid	3.0	NG	2280	10	25	NG	100	Gode et al. (2008)
Acacia	Sawdust	NaOH	6.0	NG	2280	10	NG	NG	100	Meena et al. (2008)
*Seed shells*										
Almonds	Seed shells	NG	2.0	10–100	60	24	50	300	99	Nasseh et al. (2016)
Groundnut	Seed shells	NG	8.0	15–100	120	2	41	120	59	Bayuo et al. (2019)
Palm kernel	Seed shells	NG	2.0	10–100	45	0.5	40	NG	100	Mehr et al. (2019)
Peanut	Seed shells	NG	2.0	NG	360	0.2	40	130	NG	Ahmad et al. (2017)
Groundnut	Seed shells	NG	2.0	NG	60	1–50	40	200	NG	Qaiser et al. (2009)
Peanut	Seed shells	NG	2.0	5–500	60	15	25	250	99	Mahajan and Sud (2011)
Chestnut	Seed shells	NG	2.0	100–150	360	NG	25	250	NG	Ertaş and Öztörk, 2011
Almond	Seed shells	NG	1.8	50–250	360	2.4	NG	NG	90.2	Manfe et al. (2012)
Apricot + Almond	Seed shells	NG	2.0	0.5–5	30	10–60	25	400	NG	Khazaei et al. (2011)
Almond Green	Seed shells	NG	6.0	10–50	30	4	25	45	94.1	Ahmadpour (2011)
Almond	Seed shells	NG	2.0	0.5–5	30	1–6	25	400	75.9	Aliabadi et al. (2012)
Coconut	Seed shells	NG	2.0	NG	60	8	NG	NG	86	Dave et al. (2012)
Apricot	Seed shells	NG	2.5	0.5–4	30	0.1–4	25	400	90	Aliabadi et al. (2012)
Almond	Seed shells	H_3_PO_4_	2.0	50–1,000	240	2.5	35	150	100	Rai et al. (2018)
Nutshells	Seed shells	ZnCl_2_	2.0	10–25	60	0.05	30	150	99	Kumar and Jena (2017)
Walnut	Seed shells	Citric acid	2.0	NG	120	NG	25	NG	75	Altun and Pehlivan (2012)
Almond	Seed shells	NG	2.0	60–100	70	5–25	NG	100	98	Bhatti et al. (2008)
*Leaves*										
*Sambucus nigra* L.	Leaves	NG	2.0	10	35	3	70	NG	98.22	Mancilla et al. (2023)
Jackfruit	Leaves	NG	8	NG	120	0.5	NG	150	95	Sangeetha et al. (2023)
*Euclea schimperi*	Leaves	NG	2.0	5–20	120	3	NG	NG	97.39	Gebrehawaria et al. (2015)
Green Moringa tea	Leaves	NG	2.0	10–150	60	8	60	165	99	Timbo et al. (2017)
*Cassia fistula*	Leaves	NG	2.0	NG	360	0.2	NG	130	NG	Ahmad et al. (2017)
Pistachio	Leaves	NG	2.5	0.5–4	30	0.1–4	25	400	97.1	Aliabadi et al. (2012)
*Platanus orientalis*	Leaves	NG	6–7	2	120	1	24	300	85	Mahvi et al. (2007)
Bhringraj	Leaves	NG	2.0	NG	230	1.5	NG	NG	96	Sekhar et al. (2012)
*Aerva lanata*	Leaves	NG	2.0	NG	170	2	NG	NG	92	Sekhar et al. (2012)
Neem	Leaves	NG	3–7	25–125	300	1–6	NG	160	94.5	Venkateswarlu et al. (2007)
*Juniperus procera*	Leaves	Diethyl ether and H_2_SO_4_	4.0	50–300	120	2	40	150	96	Ali et al. (2019)
*Typha elephantina*	Leaves	NG	2.0	150–400	360	10	25	180	78	Moniruzzaman et al. (2017)
*Ziziphus jujuba*	Leaves	NG	6.0	50–300	105	15	25	200	NG	Vikal et al. (2013)
Tendu	Leaves	NG	2.0	50–250	120	0.5–2	30	250	95.2	Mane (2010)
*Bark*										
*Pithecellobium dulce*	Bark	NG	5.0	250–1,000	120	1	NG	150	54	Muthulakshmi and Baskaran (2017)
*Acacia albida*	Bark	NG	2.0	5–20	60	3	NG	NG	98.47	Gebrehawaria et al. (2015)
*Pinus roxburghii*	Bark	NG	3.0	10–100	60	0.2–2	30	NG	96.2	Ahmad et al. (2005)
*Mangifera indica*	Bark	NG	NG	50–200	25	0.2–0.8	NG	NG	95	Manjusha (2012)
*Eucalyptus tereticornis*	Bark	NG	5.0	NG	NG	2	NG	NG	94	Sharma and Goyal (2011)
Eucalyptus	Bark	NG	2.0	200	60	NG	32	NG	92	Logeswari (2013)
Neem	Bark	H_2_SO_4_	NG	50–100	NG	NG	NG	NG	NG	Maheshwari and Gupta (2016)
*Lantana camara*	Bark	HNO_3_	2.0	50–100	30	4	60	NG	98	Ravulapalli and Kunta (2018)

NG: not given.

### 2.2 Tea waste

Dave et al. (2012) showed the Cr(VI) removal potential of tea waste in a batch experiment; here, the equilibrium was established at 60 min when using 8 g of the adsorbent, and the highest uptake capacity was reported as 34.25 mg g^−1^ at a low pH of 2. Furthermore, the data were best fitted to the Lagergren equation. Dizadji et al. (2011) also reported the use of brewed tea waste for adsorption of Cr(VI), where the metal uptake was reportedly maximum at a pH of 2 (adsorption capacity = 19 mg g^−1^); this confirmed the electrostatic attraction mechanism for Cr(VI) adsorption. The experimental data followed pseudo-second-order kinetics with *R* ^2^ > 0.99, showing chemisorption and formation of strong covalent bonds between the adsorbent and adsorbate. Attia (2012) compared decolorized cooking tea waste (CTW) with commercially available activated carbon (AC) for Cr(VI) treatment in batch experiments. Both adsorbents were found to be highly dependent on the pH, with the maximum adsorption (85.6 mg g^−1^ for AC and 89.24 mg g^−1^ for CTW) occurring at a low pH of 2.0. At a pH of 12, the percentage efficiencies decreased to 18 and 11 mg g^−1^ for AC and CTW, respectively, because of competition between the hydroxyl and chromate anions. Equilibrium was established at 3 h, and the data were better fitted to the Freundlich adsorption model than Langmuir, with *R* ^2^ values of 0.974 and 0.991 for CTW and AC, respectively. Ion diffusion was the dominant mechanism in the adsorption process. At 20°C, 30°C, and 40°C, Cr(VI) removal by AC were 74%, 73%, and 72.9% and that by CTW were 81.2%, 74.4%, and 73%, respectively. At an adsorbent dose of 0.9 g L^−1^ and 50 mg L^−1^of Cr(VI), the Cr(VI) treatment efficiency with AC was 99.4% and that with CTW was 99.5%. The percentage efficiencies of Cr(VI) with AC and CTW reduced from 90.6% to 50.4% and 94.8% to 53.6%, respectively, when Cr(VI) increased from 25 to 300 mg L^−1^; however, the metal uptake capacities of AC and CTW increased from 4.5 to 30.26 mg g^−1^ and from 4.74 to 32.16 mg g^−1^, respectively. The efficiencies decreased owing to less diffusion of ions. Both adsorbents followed pseudo-second-order kinetics, where CTW was more efficient than AC (Attia, 2012). Ahalya et al. (2010) investigated the potential of coffee husk as an alternative material for Cr(VI) adsorption; the experimental findings were fitted well to both the Langmuir and Freundlich isotherms; the Cr(VI) uptake capacity calculated for the Langmuir model was 44.95 mg g^−1^. Infrared spectral studies confirmed that the hydroxyl and carboxyl functional groups on the surface of coffee husk participated in the adsorption of Cr(VI) (Yadav et al., 2013).

Coffee pulp (Castilla variety) was also investigated and showed up to 87.94% removal of Cr(VI), where the point of zero charge (PZC) was found to be 3.95. The experimental data confirmed monolayer adsorption of the adsorbate. Fourier-transform infrared (FTIR) spectroscopy confirmed the presence of OH, CH, CH_2_, C=O, and C-O-C bonds associated with the adsorption mechanism. Coffee pulp was thus concluded to be an economical solution for wastewater treatment because of its low cost of implementation and maintenance without sludge production (Gómez Aguilar et al., 2019). Coffee grounds and mixed tea waste were also explored as alternatives for sequestration of Cr(VI); here, FTIR studies revealed the involvement of carbon and oxygen functional groups in both adsorbents. The maximum adsorption capacities of coffee grounds and mixed tea waste were 87.72 mg g^−1^ and 94.34 mg g^−1^, respectively. Both biomasses can be reused up to four times with more than 50% reusable efficiency. The efficiency of mixed tea waste was also assessed in a packed reactor, which revealed a breakthrough time of 30 min for 100 mg L^–1^ of Cr(VI) (Cherdchoo et al., 2019).

### 2.3 Dry shells

**FIGURE 4:**
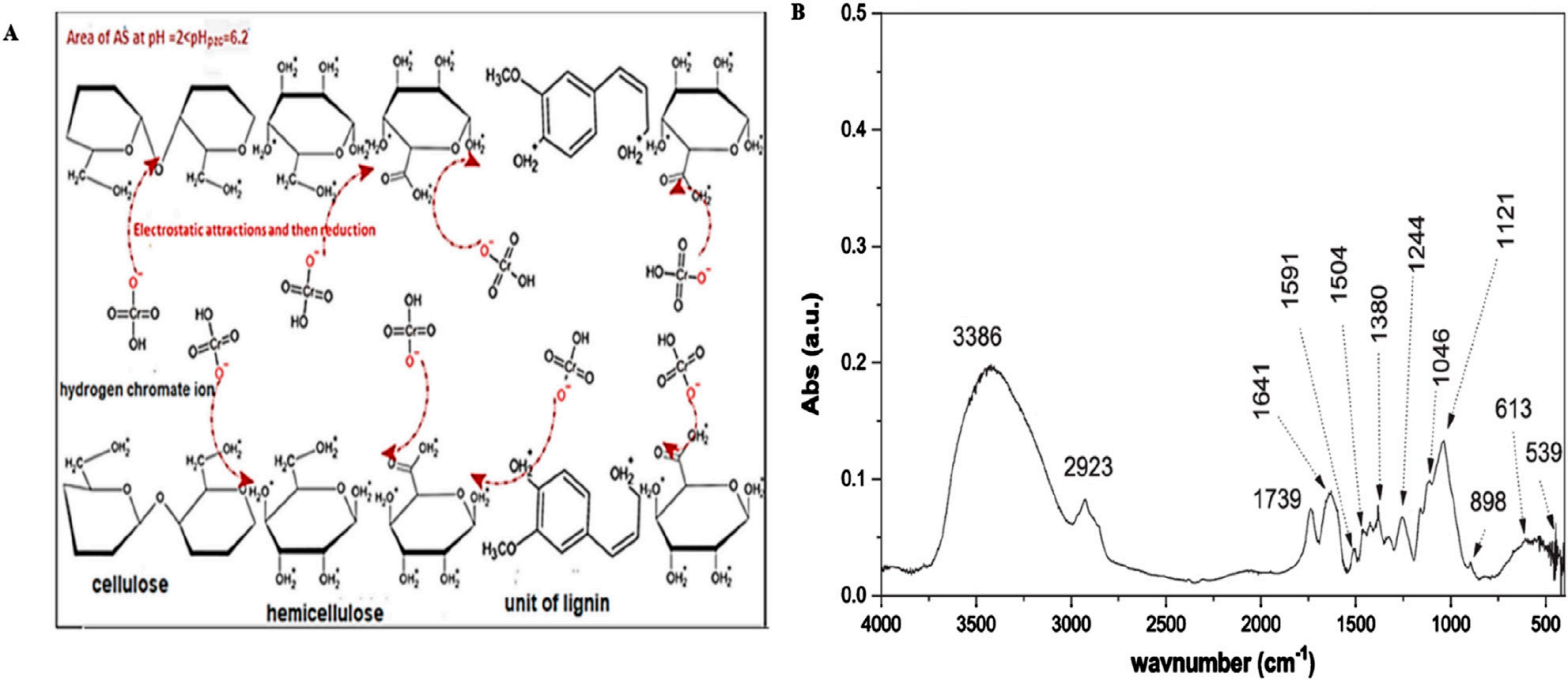
Schematic showing the **(A)** removal mechanism of Cr (VI) and **(B)** Fourier-transform infrared spectrum of almond shell (Kali et al., 2025).

Groundnuts are a type of agricultural product that is often available in bulk. Here, only the seeds are used, while the shells are wasted. The potential of groundnut shells to remove Cr(VI) has been studied by various researchers. Qaiser et al. (2009) used groundnut hulls for Cr(VI) adsorption and reported a maximum uptake capacity of 30.21 mg g^−1^. According to Mahajan and Sud (2011), *A. hypogea* shells removed almost 95% of Cr(VI) from synthetic wastewater at pH = 2; their experimental results fitted the Freundlich and Langmuir models well and followed pseudo-second-order kinetics. Ertaş and Öztürk (2011) investigated the potential of chestnut shells for removing Cr(VI) from synthetic solutions and reported an adsorption capacity of 9.47 mg g^−1^; here, the Langmuir isotherm was noted to be a better fit than the Freundlich isotherm. Cellulose, hemicellulose, and lignin are the important components of various plant and fruit shells that are responsible for Cr(VI) adsorption. The Cr(VI) removal potential of *Prunus amygdalus* (almond) shell from polluted wastewater was explored in a batch experiment (Manfe et al., 2012); this study showed that increasing the Cr(VI) solution concentration from 50 to 250 mg L^−1^ decreased the percentage removal from 80.1% to 47.1% and that increasing the adsorbent quantity from 0.8 to 2.4 g L^−1^ increased the percentage adsorption from 55.1% to 90.2%. Kali et al. (2025) observed in the case of almond shells that the maximum adsorption of Cr(VI) occurred at pH = 2 because of protonation of the shell surface (Figure 4), which indicated electrostatic attraction between the chromium anions and almond shell. Later, the electron–donor functional groups present on the almond shell surface reduced Cr(VI) to Cr(III).

Wahiba et al. (2025) used pinecone powder (PCP) for Cr(VI) adsorption and noted that Cr(VI) was removed through the binding of anionic HCrO_4_^−^ to the positively charged PCP surface as well as surface complexation. In an acidic medium, Cr(VI) ions are reduced to Cr(III) ions by the electron–donor groups on the adsorbent surface, where the acidic pH provides a large number of H^+^ sites for the reduction of Cr(VI). Khazaei et al. (2011) noted that Cr(VI) removal increased directly with the adsorbent dose when using almond and apricot shells as the adsorbents because of the increased availability of binding sites on the biomass surface. The results of batch experiments revealed that the maximum adsorption occurred at a low pH of 2. Specifically, at this pH, the Cr compounds form more HCrO_4_^−^, which are strongly attracted to the active sites. Aliabadi et al. (2012) also reported the use of almond shells for Cr(VI) treatment in a batch reactor; they recorded a high uptake at pH 2.0 and 30 min of contact time. The data were well fitted to the Freundlich isotherm and followed second-order rate kinetics. The adsorbent active sites acquired positive charges because of the excess hydrogen ions at pH 2.0, leading to strong attractions between HCrO^−^_4_ and the positively charged sites.

Almond nut shells showed 90% removal at a pH of 1.8, while almond green hulls offer the best removal of 99.6% at pH = 2 and 94% at pH = 6. This reduction in adsorption is because of the lower electrostatic attraction between the adsorbent and adsorbate. Different functional groups like amine, carboxyl, and hydroxyl groups are present on the surface of almond green hulls, which are responsible for Cr(VI) removal (Sahranavard et al., 2011). Almond green hull powder showed a maximum adsorption capacity of 10.123 mg g^−1^ at a pH of 2. The Dubinin–Raduskevich (D-R) model revealed that Cr(VI) adsorption was physical in nature as the value of E is less than 8 kJ mol^–1^ (Nasseh et al., 2016). Groundnut shells also showed maximum adsorption at optimum time, adsorbent dosage, pH, and temperature values of 120 min, 2 g L^–1^, 8, and 41.5°C, respectively. Among the four tested isotherm models, the adsorption data were best fitted to the Temkin model. The FTIR spectra revealed that –OH and –C–O groups were involved in the adsorption on groundnut shells (Bayuo et al., 2019). The adsorption potential of palm kernel shell was also investigated, and it was observed that the adsorption increased with decrease in pH and that the maximum adsorption was attained at a pH of 2. The BET surface area was reported to be 121.82 m^2^ g^−1^, and the adsorption data were well fitted to the Freundlich isotherm with a maximum adsorption capacity of 125 mg g^−1^ (Mehr et al., 2019). *A. hypogea* shells also revealed a monolayer adsorption capacity of 4.32 mg g^−1^, where the surface area and pore volume were found to be 1.78 m^2^ g^−1^ and 0.003 cm^3^ g^−1^, respectively (Ahmad et al., 2017).

### 2.4 Leaves

The leaves of various trees constitute the bulk of plant waste that can be used for the removal of different contaminants. The leaves of the mulberry, acacia, poplar, and other trees contain cellulose and hemicelluloses that are effective for adsorbing different pollutants from wastewater. Aliabadi et al. (2012) developed adsorbents from the leaves of the apricot and pistachio trees and tested their potential for percentage of Cr(VI) adsorption; this study revealed maximum Cr(VI) adsorption potentials of 97.1 mg g^−1^ at a pH of 2.0 for pistachio and 90.1 mg g^−1^ for apricot tree leaves in approximately 30 min. At acidic pH, the H^+^ ions present on the surface of the adsorbent increase, resulting in strong attractive forces between the positively charged adsorbent surface and chromate ions. At pH greater than 6.0, there is competition between the chromate and OH^−^ anions for adsorption on the adsorbent surface, which the hydroxyl ions tend to dominate, thus decreasing adsorption. The experimental data were fitted well to the Freundlich equation compared to the Temkin and Langmuir equations. Sekhar et al. (2012) reported the Cr(VI) removal potentials of the leaf powders of Bhringraj, *Aerva lanata*, and *Trileafa portulacastrum* as 96.0%, 92.0%, and 84.0%, respectively, where the equilibrium was established in 3.5 h. Lignocellulosic materials like leaves have ^−^OH/^−^COOH surface functional groups whose dissociations depend on the pH. Their surfaces are negatively charged at high pH and attract cations, whereas the surfaces are positively charged at low pH due to protonation and show affinities toward weak anions. Moringa leaf powder was shown to remove Cr(VI) by up to 90% at an equilibrium time of 90 min (Madhuranthakam et al., 2021). *Aegle marmelos* leaves also showed almost 86.6% removal of Cr(VI) at a pH of 2 (Mathai et al., 2022). Reddy et al. (2012) explored the potentials of the leaves and ashes of *Tephrosia purpurea* and *Solanum nigrum* for Cr(VI) removal from a synthetic solution in a batch experiment at low pH. Here, the physical activation was achieved at 105°C in an oven, following which 84.0% and 88.0% extractions of Cr(VI) were observed with the leaf powder and ash of *T. purpurea*, respectively, along with 100% removal with both the leaf powder and ash of *S. nigrum*. Saueprasearsit (2011) investigated durian peel for Cr(VI) removal and concluded that the optimum conditions for maximum Cr removal (10.67 mg g^−1^) were pH of 2, Cr(VI) solution concentration of 75 mg L^−1^, and contact time of 30 min. The Langmuir model and second-order kinetics were best fitted to the experimental results when desorption was carried out using 1M of HCl. Gopalakrishnan et al. (2013) reported the use of neem (*Azadirachta indica*) leaf powder for Cr(VI) adsorption; their data best followed the Freundlich adsorption isotherm, and the equilibrium time was 3 h. Neem leaves contain polar groups like –NH_2_, –COOH, and –OH, which act as adsorption sites for the adsorbate. Haleem and Abdulgafoor (2010) analyzed the utility of date palm fibers (leaf) for Cr(VI) adsorption in terms of different parameters like the metal solution concentration, pH, sorbent amount, and contact time; they found that the process was pH-dependent and that the data were best fitted to the Langmuir isotherm with a correlation coefficient of 0.908. Their results showed 98.7% efficiency for Cr(VI) removal using 5 g of the biomass, i.e., date palm leaves.

**FIGURE 5:**
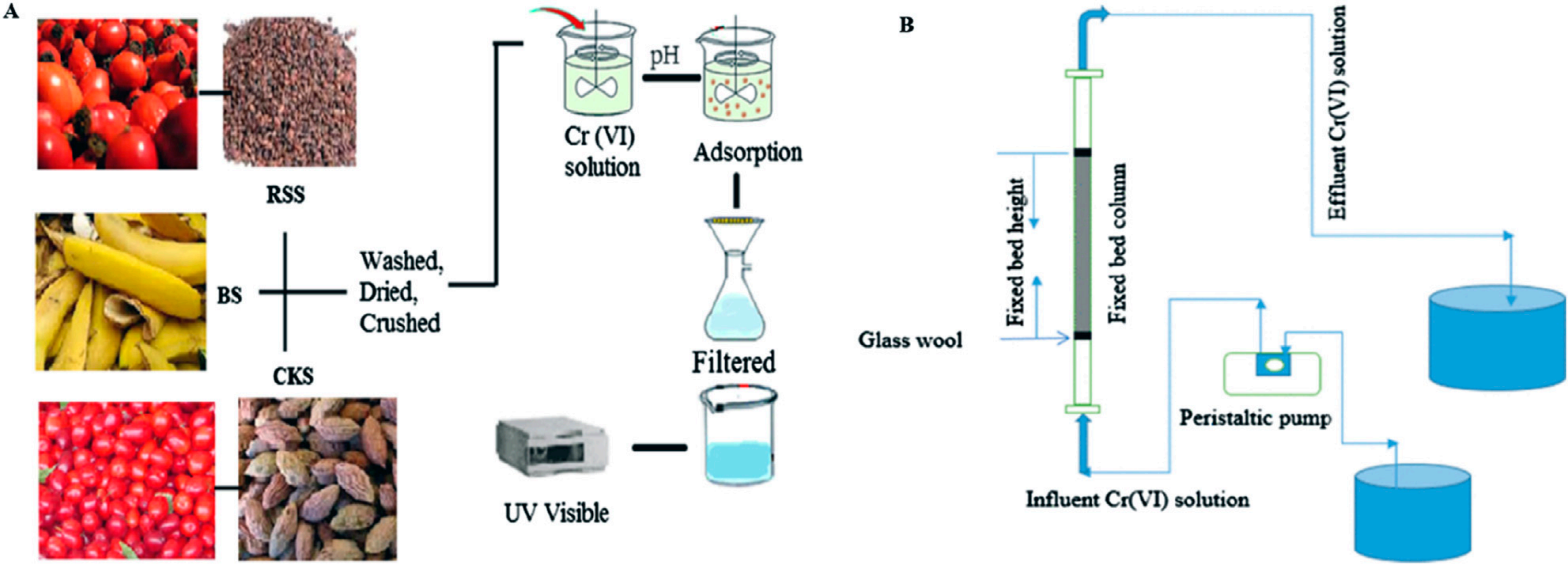
**(A)** Batch and **(B)** column experiment setups for the adsorption studies (Parlayici and Pehlivan, 2019; Abdu et al., 2024).

Pine leaves, pine needles, and pine sawdust have also been used for Cr treatment in batch and column studies (Aliabadi et al., 2006; Hadjmohammadi et al., 2011) (Figure 5). Powdered rubber leaf was also explored for Cr(VI) removal potential in batch and fixed column studies. The important functional groups involved in adsorption were amines, alkenes, carboxyl, and aromatic acids. The exhausted adsorbent was regenerated using 0.5 N NaOH, and the percentage removal of Cr(VI) by the regenerated rubber leaf powder dropped to 91.57% compared to 97.0% with the fresh adsorbent. The regenerated adsorbent could be used up to three times, but the adsorption capacity reduced to 79.4% at the end of the third cycle. The column study revealed that as the flow rate increased from 5 to 20 mL min^−1^, the percentage removal decreased from 100% to 26.89%; this is attributed to the low retention time of the adsorbate with the adsorbent. The experimental data were best fitted to the Yoon–Nelson model than the Thomas model (Nag et al., 2016).

Both raw and sulfuric-acid-modified leaves of *Ruellia patula* Jacq have been evaluated for their removal efficiencies of Cr(VI); here, the Langmuir adsorption capacity was higher for the modified adsorbent (62.50 mg g^−1^) than the raw form (37.03 mg g^−1^). Desorption was carried out using sodium hydroxide and offered the best results for the first three cycles (Saranya et al., 2017). Okra leaves have also been investigated for Cr(VI) removal, where the maximum adsorption capacity of 81.94 mg g^−1^ was observed at pH = 2. From a sample of industrial wastewater, okra leaves were noted to adsorb approximately 92.15% of Cr. A regeneration study was also conducted using different chemicals, which showed that 96% of the adsorbent ions could be recovered using up to 5 mL of 1M HCl solution. An interference study revealed that in the presence of Na_2_C_2_O_4_, Cr(VI) adsorption increased by up to 6%. FTIR studies have specified the involvement of hydroxyl, oxime, and carboxylic acid groups in adsorption (Khaskheli et al., 2016). Waste material like the leaves of green tea have also shown the highest percentage removal (99%) of Cr(VI). A desorption study showed that 0.1 N HCl had the best performance among four different solutions. Jeyaseelan and Gupta (2016) showed through an interference study that Cd and Zn allow increased adsorption of Cr(VI) owing to their increased surface areas, whereas Cu, Ni, and Fe decrease Cr(VI) adsorption through competition among the various ions.

*E. schimperi* leaves showed a maximum adsorption of 97.39% at an optimum pH of 2, where the Langmuir isotherm best described the adsorption of Cr(VI) (Gebrehawaria et al., 2015). Green moringa tea leaves were used to accomplish a Cr(VI) uptake capacity of 33.9 mg g^−1^ at a pH of 2 with an initial adsorbate concentration of 100 mg L^−1^; here, the equilibrium data were found to be best fitted to the Freundlich isotherm (Timbo et al., 2017). *C. fistula* leaves were found to have a maximum monolayer adsorption of 4.48 mg g^−1^ at an equilibrium time of 360 min, where the surface area of the leaves was 1.09 m^2^ g^−1^ (Ahmad et al., 2017). FTIR spectrum of the *Typha elephantina* Roxb (Hogla) leaves indicates that stretching of the OH, O=C=O, C–H, and C=C bonds are responsible for Cr(VI) removal; treatment of industrial effluents with hogla leaves was reported to decrease adsorption from 78.7% to 44.8% owing to the presence of other contaminants (dyes and metal ions) in addition to Cr(VI) in the effluent (Moniruzzaman et al., 2017).

### 2.5 Agricultural waste

Naiya et al. (2011) investigated different low-cost agricultural wastes like rice straw, bran, and husk for Cr(VI) adsorption. Characterization of the raw and Cr(VI)-loaded adsorbents by FTIR spectroscopy revealed the presence of hydroxyl, alkene, aromatic, nitro, silicon oxide, and carboxylate anion groups on the adsorbents that were responsible for the uptake of metals from wastewater. Biomass-based natural waste materials follow the complex process of Cr(VI) adsorption through several mechanisms, including electrostatic attraction, reduction, and ion exchange (Assefa et al., 2024). Agricultural waste like crop residues undergo ion exchange through the interactions of various functional groups (amine, hydroxyl, and carboxyl) on the adsorbent surface with the Cr(VI) anions. The solution pH plays a critical role in the exchange of these functional group cations with Cr(VI) anions (Njoya et al., 2021). The surfaces of organic waste become more attractive to Cr(VI) anions at acidic pH values owing to protonation, as observed by Gning et al. (2024); however, deprotonation of the adsorbent functional groups occurs at basic or higher pH values, resulting in reduced ion exchange (Xie et al., 2021).

**FIGURE 6:**
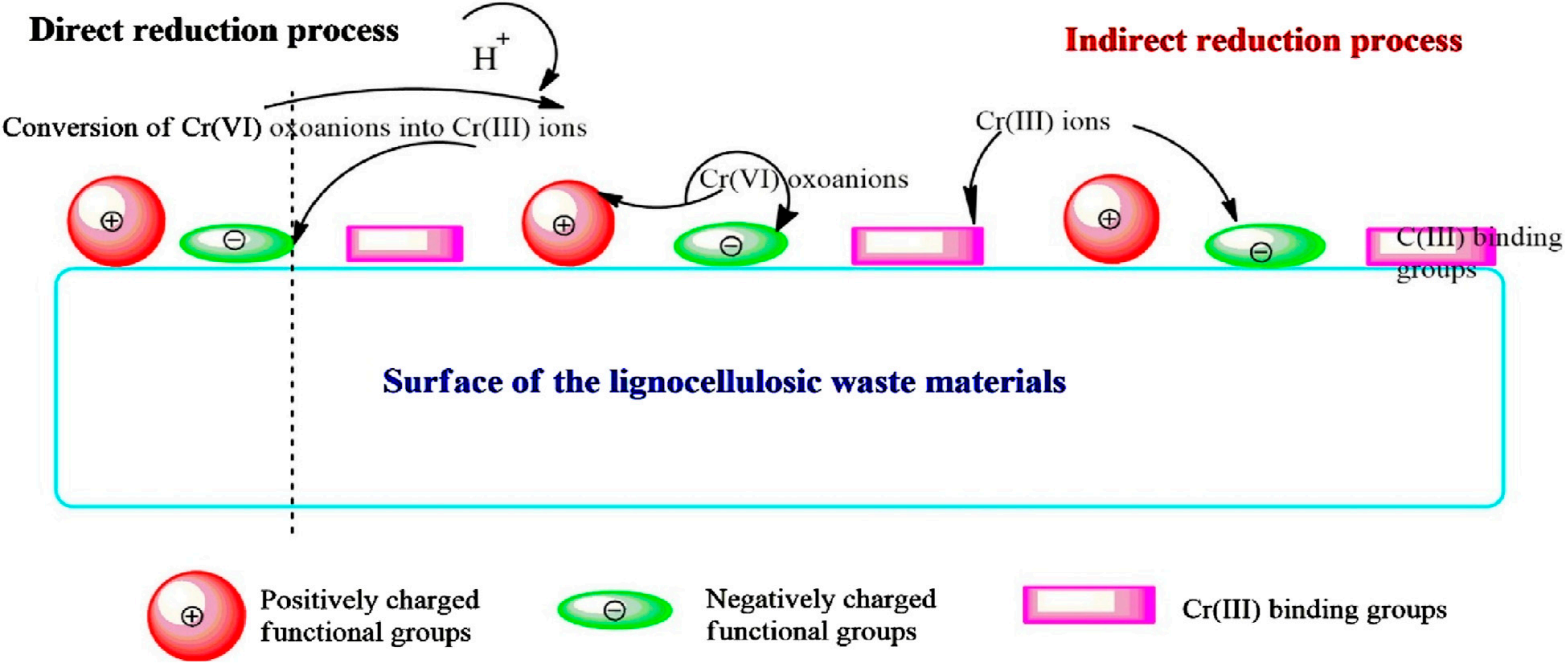
Possible mechanisms of Cr(VI) adsorption (Basnet et al., 2022).

Cr(VI) can be reduced to Cr(III) by direct or indirect reduction, as shown in Figure 6. When the pH is low, direct reduction of Cr(VI) to Cr(III) is observed because of its higher reduction potential than the functional groups present on the adsorbent surface, which results in bond formation of Cr(III) with the adsorbent functional groups. On the other hand, three steps are involved in indirect reduction. First, oxoanions of Cr(VI) are adsorbed on the protonated functional groups like amino and carboxyl groups; second, Cr(VI) is reduced to Cr(III) with the help of the electron-donating functional groups on the adsorbent surface; third, Cr(III) forms complexes with the adsorbent functional groups or is repulsed by the electron-rich groups on the adsorbent surface and is released into the solution (Basnet et al., 2022). Under acidic conditions, adsorption coupled with reduction was observed for Cr(VI) in the presence of *Prosopis cineraria* leaf powder (Soleimani et al., 2024).

Rice husk has been employed to remove Cr(VI) and has shown almost 78.6% removal efficiency at a pH of 5.2 (Khalil et al., 2021). Maize biomass can also be used as an adsorbent; maize as a grain is used as food, whereas the other parts of the plant are used as fodder for livestock. The tassels and cobs of maize are abundantly available in developing countries and are economical alternatives for Cr adsorption. Murugesan et al. (2012) tested the potentials of acid-modified corn cob (AMCAC) and unmodified corn cob (UMCAC) for Cr(VI) adsorption. At lower metal-ion concentrations, 96% adsorption was observed, which is attributed to the interactions between all metallic ions and the active sites of the adsorbent. At higher concentrations of the metal ions, adsorption decreases as the metals compete for available active sites on the biomass surface, resulting in saturation of the binding sites; in this case, 95% removal was observed at the optimum time of 60 min. High amounts of the adsorbent (25–100 mg) can increase the removal of Cr ions from 26.22% to 90.06% because of the increased number of exchangeable binding sites. Here, the optimum adsorbent dose was reported to be 100 mg; the equilibrium data were best fitted to the Redlich–Peterson model and followed pseudo-second-order kinetics. The maximum metal uptake capacities were 22.82 mg g^−1^ and 54.11 mg g^−1^ for UMCAC and AMCAC, respectively. Hydroxyl, aldehyde, and halide groups are present on the biomass surface and are responsible for Cr(VI) adsorption (Murugesan et al., 2012). Mahajan and Sud (2013) studied three different forms of *Acacia saligna* pods (lignocellulosic nitrogenous waste), namely *A. saligna* pods natural (ASPN), *A. saligna* pods carbon (ASPC) in the AC form, and *A. saligna* pods beads (ASPBs) impregnated in hydrated beads form, for the batch treatment of Cr(VI). Here, the FTIR spectrum reveals that various functional moieties like C-H, hydroxyl, –C=O, and –OCH_3_ groups are responsible for Cr(VI) adsorption on *A. saligna* pods. The data follow second-order kinetics with good correlation coefficients, and the greatest removal was achieved at a low pH of 2. The efficiency of removal was observed in the following order: ASPC (97%) > ASPBs (94%) > ASPN (92%). The equilibrium condition was attained at 30 min for ASPC as well as 60 min for ASPBs and ASPN. Thus, it was concluded that ASPBs possessed more removal efficiency than natural forms of the pods (Mahajan and Sud, 2013). Singha et al. (2011) explored the Cr batch removal efficiencies of naturally available adsorbents, including rice straw, rice bran, hyacinth roots, neem bark, and neem leaves. The FTIR spectra revealed the presence of various functional moieties like hydroxyl, aromatic, alkene, silicon oxide, carboxylate, nitro, and sulfonic acid groups, which were responsible for Cr(VI) adsorption. The maximum adsorption was attained at 10 g L^−1^ of the adsorbent amount in 3–6 h. The experimental data were best fitted to the Langmuir isotherm, and the sorption energy was obtained from the D-R model, which showed the involvement of a chemisorption-based adsorption process (Singha et al., 2011). Manjusha (2012) examined the utility of papaya peel powder and observed higher Cr(VI) removal at lower initial metal-ion concentrations in wastewater.

The novel adsorbent *Artemisia absinthium* showed that Cr(VI) treatment efficiency decreases in the presence of an electrolyte such as 0.1 N potassium nitrate; the monolayer capacity was reportedly 46.99 mg g^−1^. According to the intraparticle diffusion model, Cr(VI) adsorption occurs in three stages; further, approximately 93% of Cr(VI) can be recovered from the column using 0.01 N sodium hydroxide (Rao et al., 2015). A previous report showed that approximately 1 g of *Caryota urens* inflorescence waste as an adsorbent biomass can treat approximately 175 mL of Cr(VI) solution (Rangabhashiyam and Selvaraju, 2015). Ash gourd peel waste having a BET surface area of 0.4854 m^2^ g^−1^ was found to be effective for Cr(VI) adsorption (18.7 mg g^−1^); according to the BDST, the uptake capacity was found to be 128.98 mg g^−1^ (Sreenivas et al., 2014).

Grass pea is a common food legume and is an agricultural waste. Chakravarty et al. (2013) investigated the use of *Lathyrus sativus* husk (grass pea or chickling vetch) for Cr(VI) adsorption; the results of this study displayed that the optimum pH was 2.0 and optimum time was 90 min. The FTIR data reflected the presence of different functional groups like -NH_2_, -COOH, -OH, and -PO_4_^3−^, which were responsible for Cr adsorption through complexation and ion exchange mechanisms. According to Zeng et al. (2025), Cr(VI) adsorption on carbon-based organic adsorbents occurs through various mechanisms, such as electrostatic interactions, surface complexation, and ion exchange. In particular, ion exchange and electrostatic interaction mechanisms were reported to be dominant in acidic pH along with carbon-based adsorbents (Sinha et al., 2022). Logeswari et al. (2013) investigated low-cost biomaterials like eucalyptus bark (EB) and rice husk for Cr(VI) removal, where the maximum uptake of Cr(VI) was observed to be 45 mg g^-1^ at a low pH of 2 and an equilibrium time of 2 h. The maximum removal efficiency of 92% was achieved with EB, while rice husk showed an efficiency of only 26%; this difference in the percentage removal efficiencies of different adsorbents is attributable to the different functional groups present on the adsorbents for binding Cr(VI) (Logeswari et al., 2013). The seeds of *Phyllanthus acidus* (gooseberry) enabled 96% Cr(VI) removal under an acidic pH of 2 and equilibrium time of 1 h; the adsorption was confirmed to be monolayer and had a Langmuir *R* ^2^ of 0.992 (Aravind et al., 2015).

### 2.6 Bark

Sharma and Goyal (2011) showed that the bark of *Eucalyptus tereticornis* had removal efficiencies of 70% and 94% for Cr(VI) present in tannery effluents and chrome-plating effluent, respectively. Manjusha (2012) also reported the use of the ecofriendly material *Mangifera indica* bark dust for Cr(VI) removal in a batch system, where the optimum adsorbent dosage was 0.8 g L^−1^ and optimum time was 25 min; the Cr(VI) removal was observed to follow a smooth curve, indicating monolayer coverage. The bark of *Pithecellobium dulce* was investigated for Cr(VI) removal, and it was found that maximum Cr(VI) removal was obtained at a pH of 5 with an adsorbent dose of 1 g L^−1^ (Muthulakshmi and Baskaran, 2017). *A. albida* bark was shown to have a Cr(VI) removal efficiency of up to 98.47% at an optimum pH of 2; here, the equilibrium data followed the Langmuir isotherm and pseudo-second-order kinetics, and the FTIR spectrum revealed the presence of polar functional groups on the adsorbent surface like hydroxyl, amide, and amine groups for Cr(VI) adsorption (Gebrehawaria et al., 2015). The optimum conditions for Cr(VI) removal using a fixed-bed column containing sago bark (*Metroxylon sagu*) were a flow rate of 2 mL min^−1^ and bed depth of 9 cm; it was also found that used sago bark could be regenerated using 0.01 M of nitric acid (Fauzia et al., 2021).

### 2.7 Microbial biomasses

#### 2.7.1 Algae

Raw and chemically modified *Spirulina platensis* were reported to have adsorption capacities of 79.6 and 158.7 mg g^−1^, respectively, in a batch reaction. A continuous column study with the modified adsorbent confirmed maximum adsorption capacity at a flow rate of 50 mL h^−1^ and an initial metal concentration of 200 mg L^−1^. Both the raw and modified algae could be regenerated by up to 89.6% and 94.3%, respectively (Bayramoglu et al., 2016). Blue–green marine algae have been reported to have a maximum adsorption capacity of 37.426 mg g^−1^ under optimum time and agitation speed conditions (Ramadoss and Subramaniam, 2018). *Sargassum myriocystum* is a marine brown alga that has been explored for Cr(VI) removal potential; it has been shown to have sulfate groups on the surface along with hydroxyl, carboxylic, aldehyde, and carbonyl groups that are involved in Cr(VI) adsorption. Economic analysis of treatment with *S. myriocystum* revealed 60% lower cost for chromium effluents compared to activated charcoal treatment (Jayakumar et al., 2015). *Pseudopediastrum boryanum* var. *longicorne* also displayed maximum removal at an adsorbent dose of 2 g L^−1^ and initial concentration of 10 mg L^−1^ (Sutkowy and Kłosowski, 2018). Three types of algae were evaluated for Cr(VI) removal, and the results showed that *Cladophora glomerata* was a better adsorbent than *Enteromorpha intestinalis* and *Microspora amoena*; increase in the adsorbent dose beyond 1 g resulted in decreased metal removal, which may be attributed to large block formation of the adsorbent particles resulting in lower available surface area for Cr(VI) removal (Al-Homaidan et al., 2018).

*Pithophora oedogonia* contains carbonyl, carboxyl, and hydroxyl functional groups that are responsible for 90% Cr(VI) removal at pH = 4 (Suleman et al., 2017). *Chlorella vulgaris* green micro algae was explored to be an economical and efficient adsorbent for Cr(VI) adsorption of up to 99.75% (Indhumathi et al., 2014). The economical green algae *Ulva fasciata* sp. showed efficient removal (77%) of Cr(VI) at pH = 6 (Prasanna Kumar et al., 2017). *Polysiphonia urceolata* and *Chondrus ocellatus* were found have maximum uptake capacities of 170.6 mg g^−1^ and 113.4 mg g^−1^, respectively, at optimum conditions and obeyed pseudo-second-order kinetics reflecting a chemisorption mechanism; the results also revealed the involvement of carbonyl, hydroxyl, and amino groups in Cr(VI) adsorption (Li et al., 2015). Valli et al. (2013) explored the efficiency of green algae biomass for Cr(VI) adsorption in a batch experiment and reported 75% removal. Kurniasih et al. (2013) investigated the efficiency of immobilized algal bloom biomass for Cr(VI) removal and observed the highest percentage removal when the algal bloom biomass was treated with 0.1 N HCl; here, the maximum uptake capacity was reported to be 11.494 mg g^−1^.

Esmaeili et al. (2010) studied and compared the Cr(VI) removal efficiencies of dried (BD) *Sargassum* spp. (brown marine algae) and its AC form; the optimum pH and equilibrium time were found to be 2.0 and 120 min, respectively, while the maximum adsorption capacities was observed to be 3.69 mg g^−1^ for the BD and 6.877 mg g^−1^ for the AC forms. Sujin Jeba Kumar et al. (2013) examined the immobilized cells of a microalgae (*Isochrysis galbana*) for Cr(VI) adsorption; they reported a maximum uptake capacity of 29.21 mg g^−1^ at pH = 4 and temperature of 35°C, where the experimental data were well fitted to the Langmuir model. Table 2 presents a comparison of different algal biomasses used as adsorbents for Cr(VI) removal.

**TABLE 2:** Comparative analysis of the process variables for the adsorption of Cr(VI) using algal biomass adsorbents.

Algal species	Modified with	pH	Metal concentration (mg/L)	Time (min)	Dose (g/L)	T (^o^C)	Mixing speed (rpm)	Removal (%)	Reference
*Chlorella sorokiniana*	NG	NG	NG	4,320	NG	NG	NG	99.6	Husien et al. (2019)
*Spirulina platensis*	NG	3.0	NG	NG	2	45	NG	94.2	Bayramoglu et al. (2016)
*Spirulina platensis*	4-Aminopyridine	3.0	NG	NG	2	45	NG	98.7	Bayramoglu et al. (2016)
Blue-green marine algae	NG	5.0	2–250	1,440	2	NG	120	NG	Ramadoss and Subramaniam (2018)
*Sargassum myriocystum*	NG	5.2	50–250	108	2	NG	120	85	Jayakumar et al. (2015)
*Pseudopediastrum boryanum* var. *longicorne*	NG	2.0	10–100	NG		NG	250	70	Sutkowy and Kłosowski (2018)
*Cladophora glomerata*	NG	2.0	10–20	60	1	45	200	66	Al-Homaidan et al. (2018)
*Enteromorpha intestinalis*	NG	2.0	10–20	60	1	45	200	48	Al-Homaidan et al. (2018)
*Microspora amoena*	NG	2.0	10–20	60	1	45	200	53	Al-Homaidan et al. (2018)
*Pithophora oedogonia*	NG	4.0	NG	NG	1	30	180	90	Suleman et al. (2017)
*Chlorella vulgaris*	NG	3.0	20–120	120	0.12	NG	120	99.75	Indhumathi et al. (2014)
*Ulva fasciata* sp.	NG	6.0	20–100	30	3	NG	180	77	Prasanna Kumar et al. (2017)
*Polysiphonia urceolata*	NG	2.0	25–250	60	4	45	150	>65	Li et al. (2015)
*Chondrus ocellatus*	NG	2.0	25–250	40	4	45	150	>55	Li et al. (2015)
*Spirulina platensis*	H_2_SO_4_	3.0	50–750	30	1	30	80	99.81	Gunasundari and Kumar (2016)
*Padina boergesenli*	NG	1.0	50–350	180	4	NG	150	100	Thirunavukkarasu and Palanivelu (2007)
Green algae	NG	2.0	50–80	30	5	NG	150	75	Valli et al. (2013)
*Laminaria japonica*	NG	1.0	100–500	540	4	30	NG	60	Wang et al. (2008)
*Porphyra yezoensis* Ueda	NG	1.0	100–500	540	4	30	NG	60	Wang et al. (2008)
*Oedogonium hatei*	NG	2.0	50–100	110	0.8	45	NG	NG	Gupta and Rastogi (2009)
*Ulva lactuca*	NG	1.0	5–250	120	2–15	25	200	92	El-Sikaily et al. (2007)

NG: not given.

#### 2.7.2 Bacteria

Deepali (2011) explored Cr(VI) treatment using three bacterial strains, namely *Pseudomonas putida*, *Pseudomonas aeruginosa,* and *Bacillus* sp. At an initial Cr(VI) concentration of 10 mg L^−1^, the maximum Cr(VI) removal was observed to be 75% by *Bacillus* sp., while *P. aeruginosa* showed 69.70% removal at a concentration of 40 mg L^−1^. However, over a duration of 96 h, *P. putida* showed maximum removal of 90.88% at an initial Cr(VI) concentration of 10 mg L^−1^; this bacterium also showed maximum Cr(VI) removal at lower initial concentrations than the other two bacteria (Deepali, 2011). Karthikeyan and Mythili (2011) investigated the potential of *Bacillus* sp. and *Staphylococcus* sp. for the adsorption of Cr(VI); for *Bacillus* sp., the optimum temperature and pH were found to be 37°C and 7, respectively; however, for *Staphylococcus* sp., the optimum temperature and pH were found to be 37°C and 8, respectively. Table 3 presents a comparison of various bacterial biomasses used as adsorbents for Cr(VI) removal.

**TABLE 3:** Comparative analysis of the process variables for the adsorption of Cr(VI) using bacterial biomass adsorbents.

Bacterial species	Modified with	pH	Metal concentration (mg/L)	Time (min)	Dose (g/L)	T (^o^C)	Mixing speed (rpm)	Removal (%)	Reference
*Bacillus subtilis*	NG	2.0	25–200	NG	0.2–1	NG	150	37.5	Sethuraman and Balasubramanian (2010)
*Enterobacter cloacae*	NG	2.0	25–200	NG	0.2–2	NG	150	94.9	Sethuraman and Balasubramanian (2010)
*Pseudomonas aeruginosa*	NG	6.0	25–200	NG	0.2–3	NG	150	67.9	Sethuraman and Balasubramanian (2010)
*Pseudomonas putida*	NG	NG	10–50	5760	NG	30	120	90.88	Deepali (2011)
*Pseudomonas aeruginosa*	NG	NG	10–50	5760	NG	30	120	69.7	Deepali (2011)
*Bacillus* sp.	NG	NG	10–50	5760	NG	30	120	75	Deepali (2011)
*Pseudomonas aeruginosa*	NG	NG	NG	4320	NG	NG	NG	52.26	Chatterjee et al. (2011)
*Bacillus* spp.	NG	2.0	NG	NG	NG	37	150	95	Karthikeyan and Mythili (2011)
*Staphylococcus* spp.	NG	8.0	NG	NG	NG	37	150	97	Karthikeyan and Mythili (2011)
*Pseudomonas* sp*.* (alive)	NG	NG	NG	240	NG	NG	NG	NG	Ag and Maheswari (2007)
*Bacillus subtilis* (Dead)	NG	2.0	50–150	NG	1–3	30	NG	NG	Sivaprakash et al. (2009)
*Nostoc muscorum*	NG	3.0	NG	120	0.2–1.6	25	NG	93.2	Gupta and Rastogi (2008)
*Litinus sajor caju* (free)	NG	2.0	100	120	25	25	200	18.9	Arıca and Bayramoğlu (2005)
*Litinus sajor caju* (Immobilized)	NG	2.0	100	120	25	25	200	32.2	Arıca and Bayramoğlu (2005)
*Eriobotrya japonica*	NG	5.0	10–100	60	1–40	NG	120	68	Abuein and Belazi (2009)
*Bacillus subtilis*	NG	2.0	50–150	480	2	30	100	NG	Sivaprakash et al. (2009)
*Nostoc calcicola HH-12*	Sodium alginate	3.0	20	30	NG	26	120	70	Anjana et al. (2007)
*Chroococcus* sp.	Sodium alginate	4.0	20	30	NG	27	120	60	Anjana et al. (2007)
*Pseudomonas* sp. (dead)	NG	NG	NG	240	NG	80	NG	49.6	Ag and Maheswari (2007)
*Pseudomonas* sp. (alive)	NG	NG	NG	240	NG	80	NG	44	Ag and Maheswari (2007)
*Pseudomonas* sp.	NG	NG	NG	240	NG	80	NG	66.55	Ag and Maheswari (2007)
*Lyngbya putealis HH-15*	Sodium alginate	2–3	10–100	120	NG	25	120	82	Kiran et al. (2007)

NG: not given.

#### 2.7.3 Fungi

Abdel-Razek (2011) studied the efficiency of Ca-alginate (CA) beads as well as dead and immobilized alive *Cunninghamella elegans* beads for Cr(VI) adsorption; here, the dead beads displayed greater Cr(VI) removal efficiency than the alive and CA beads. Different fungal species obtained from contaminated samples of tannery industrial wastewater in a study area in India (Nagalkeni, Chennai) were investigated for their Cr(VI) removal potential; it was observed that at an optimum pH of 3 and adsorbent dose of 6 g, *Aspergillus niger* enabled maximum Cr(VI) removal than *Aspergillus fumigatus* and *Aspergillus flavus* (Shankar et al., 2014). *A. fumigatus* Fresenius obtained from Sukinda (India) mine water was proven to be a good adsorbent as it removed 97% Cr(VI) at an optimum pH of 5.5 and optimum time of 120 h; the FTIR spectrum confirmed the presence of hydroxyl, amine, amide, and carboxylate groups on the cell wall of the fungus; the exhausted adsorbent could also be reportedly regenerated by shaking with 0.5 M of HCl at 35°C for 2 h (Dhal and Pandey, 2018).

The adsorption capacities of both active and inactive *Pleurotus ostreatus* were evaluated, which revealed that inactive *P. ostreatus* allowed 100% removal of 50 mg L^−1^ of Cr(VI) at an optimum time of 22 min while active *P. ostreatus* enabled 100% removal of 25 mg L^−1^ of Cr(VI) at 360 h (da Rocha Ferreira et al., 2019). As a novel adsorbent, *A. niger* spores were tested for Cr(VI) adsorption capacity in a batch reactor, which revealed a maximum uptake of 97.1 mg g^−1^ at optimum conditions of temperature (40°C), dose, pH (2), and initial concentration. Interference studies of Cr(VI) with Na, K, Ca, and Mg ions showed that there was very little effect on the adsorption of Cr(VI) in the presence of these interfering ions. The proposed adsorption mechanism involved reduction of Cr(VI) to Cr(III), following which Cr(III) attached to the surfaces of the spores through electrostatic forces, complex formation, or redox reaction. The FTIR spectrum reflected that the nucleic acids and proteins of the adsorbent participated in the binding of Cr(VI) to the adsorbent surface (Ren et al., 2018).

Artist’s bracket fungus was explored for its Cr(VI) adsorption potential at various batch parameter values of the initial metal concentration, dose, and pH, which showed that the PZC value of the fungus was 3 (Pourkarim et al., 2017). *A. niger* was found to have an adsorption capacity of 11.792 mg g^–1^ at pH = 2 and follow pseudo-second-order kinetics; here, approximately 94% of the Cr(VI) was removed, and the adsorbent could be regenerated using 0.5 M EDTA; further, amines of the adsorbent cell wall were found to be involved in Cr(VI) adsorption (Mondal et al., 2017). Different species of fungi were isolated from the contaminated soil of a tannery in India, and the results revealed a maximum removal efficiency of 96.3% of Cr(VI) by *A. niger* compared to other fungal species (Sivakumar, 2016). Majumder et al. (2017) reported a maximum uptake capacity of 100.69 mg g^−1^ for *Arthrinium malaysianum*, where the used adsorbent can be regenerated by up to 41.17% using sodium hydroxide solution. Table 4 presents a comparison of various fungal biomasses used as adsorbents for Cr(VI) removal.

**TABLE 4:** Comparative analysis of the process variables for the adsorption of Cr(VI) using fungal biomass adsorbents.

Fungal species	Modified with	pH	Metal concentration (mg/L)	Time (min)	Dose (g/L)	T (^o^C)	Mixing speed (rpm)	Removal (%)	Reference
*Aspergillus niger*	NG	4.6	NG	48.45	0.05	60	500	87.8	Mondal et al. (2017)
*Aspergillus niger*	NG	3.0	NG	10,080	NG	NG	NG	96.3	Sivakumar (2016)
*Arthrinium malaysianum*	NG	3.0	NG	28,800	NG	NG	NG	67	Majumder et al. (2017)
*Aspergillus niger*	NG	3.0	NG	7,200	NG	NG	NG	93	Shankar et al. (2014)
*Aspergillus fumigatus*	NG	3.0	NG	7,200	NG	NG	NG	89	Shankar et al. (2014)
*Aspergillus flavus*	NG	3.0	NG	7,200	NG	NG	NG	84	Shankar et al. (2014)
*Aspergillus fumigatus* Fresenius	NG	3.0	NG	7,200	1	35	100	97	Dhal and Pandey (2018)
*Pleurotus ostreatus*	NG	3.0	NG	360	NG	NG	150	100	da Rocha Ferreira et al. (2019)
*Aspergillus niger spores*	NG	2.0	25–200	NG	2	40	120	NG	Ren et al. (2018)
*Litinus sajor caju* (free)	NG	2.0	100	120	25	25	200	18.9	Arıca and Bayramoğlu (2005)
*Litinus sajor caju* (immobilized)	NG	2.0	100	120	25	25	200	32.2	Arıca and Bayramoğlu (2005)
*Cunninghamella elegans* sp.	Ca alginate	2.0	25–1000	120	NG	25	150	NG	Abdel-Razek (2011)
*Phanerochaete chrysosporium*	NG	NG	100	120	NG	RT*	100	48.6	Nikazar et al. (2008)
*Penicillium canescens*	NG	6.0	100	240	NG	20	100	34.8	Say et al. (2003)
*Aspirgillus oryzai*	NG	2.0	5	330	10	37	150	89	Issac et al. (2012)
*Aspergillus sojae*	NG	2.0	5	330	10	37	150	85	Issac et al. (2012)
*Eriobotrya japonica*	NG	5.0	10–100	60	1–40	NG	120	68	Abuein and Belazi (2009)
*Aspergillus niger*	NG	4.5	NG	8,640	NG	30	200–300	92	Shugaba et al. (2012)
*Fusarium solani*	Polyethyleneimine	4.5	NG	8,640	NG	30	200–300	96	Shugaba et al. (2012)

NG: not given.

#### 2.7.4 Yeast

Yeast is a eukaryotic organism that is usually unicellular; the cytoplasm present in the living yeast cell is involved in interactions with metal ions, so yeast was tested for Cr(IV) removal potential. The carboxymethyl cellulose present in *Lentinus sajor caju* was evaluated for Cr(VI) treatment, which showed a removal capacity of 32 mg g^−1^ at a pH of 2, temperature of 25°C, and initial metal solution concentration of 100 mg L^−1^. Numerous functional groups are present of the cell wall of mycelia (COOH, =NH, -NH_2_, -OH, and -SH), which indicate the interactions of the adsorbent surface with metal ions (Arıca and Bayramoğlu, 2005). The biosorption capacity of *Kodamaea transpacifica* was found to be higher than those of *Kazachstania yasuniensis* and *Saturnispora quitensis* because of its higher surface area of 1,588.27 m^2^ L^−1^ (Campaña-Pérez et al., 2019). Table 5 presents a comparison of various types of yeasts used as adsorbents for Cr(VI) removal.

**TABLE 5:** Comparative analysis of the process variables for the adsorption of Cr(VI) using yeast-based adsorbents.

Yeast species	Modified with	pH	Metal concentration (mg/L)	Time (min)	Dose (g/L)	T (^o^C)	Mixing speed (rpm)	Removal (%)	Reference
*Candida tropicalis*	NG	2.0	20	30	0.025	30	220	87	Yin et al. (2008)
*Saccharomyces cerevisiae*	NG	6.0	204	120	NG	30	150	84	Parvathi and Nagendran (2007)
*Yarrowia lipolytica* NCIM 3589	NG	1.0	950	120	1.49	35	130	64	Bankar et al. (2009)
*Yarrowia lipolytica* NCIM 3590	NG	1.0	955	120	1.7	35	130	46	Bankar et al. (2009)
*Cunninghamella elegans* sp.	NG	2.0	25–1,000	120	NG	25	150	NG	Abdel-Razek (2011)
*Eriobotrya japonica*	NG	5.0	10–100	60	1–40	NG	120	68	Abuein and Belazi (2009)
*Saccharomyces cerevisiae*	Chitosan lignosulfate	NG	30–130	NG	1	30	180	86.95	Saifuddin and Raziah (2007)
*Kazachstania yasuniensis*	NG	4.0	10–100	10–30	NG	NG	NG	80	Campaña-Pérez et al. (2019)
*Kodamaea transpacifica*	NG	NG	10–100	10–30	NG	NG	NG	80	Campaña-Pérez et al. (2019)
*Saturnispora quitensis*	NG	NG	10–100	10–30	NG	NG	NG	80	Campaña-Pérez et al. (2019)

NG: not given.

## 3 Development of AC from waste biomasses

Pal and Kaur (2013) investigated the effects of different particle sizes (0.3–1.0 mm) of physically activated sawdust of *Dalbergia sissoo* (ASD) for Cr(VI) adsorption in a batch experiment. They noted that the Cr(VI) removal improved from 66% to 85.4% with increase in adsorbent amount from 0.2 to 1.2 g per 100 mL for ASD particles of size 0.3 mm. Moreover, improved adsorption from 61% to 81.2% was observed with a particle size of 1.0 mm. However, when the metal concentration was increased over 5–50 mg L^−1^, the efficiency of Cr removal reduced from 87% to 73% for ASD of 0.3 mm size and from 83% to 70% for ASD of 1.0 mm size. Maximum adsorption was achieved at a pH of 2.0, at which point the adsorptive surface became more positively charged and showed greater attraction to anionic species of Cr(VI). At high pH, lower adsorption was observed owing to reduced attractive forces between the anionic species of Cr and adsorbent surface. Equilibrium was established within 60 min, and there was no further adsorption thereafter because of non-availability of vacant sites. The data were best fitted to the Freundlich and Langmuir isotherms (Pal and Kaur, 2013). Physically activated *Ziziphus spina-christi* leaves showed a maximum Cr(VI) removal efficiency of 97.22% at optimum time, pH, initial metal concentration, adsorbent dose, and temperature conditions. The adsorption data were fitted well to the Langmuir isotherm, reflecting a maximum adsorption capacity of 13.81 mg g^−1^. The thermodynamic parameters also revealed the endothermic nature of Cr(VI) adsorption (Abshirini et al., 2018). Physically AC of rice straw showed a maximum Cr(VI) uptake of 90% at pH = 8. At optimum time, pH, and temperature, the rice straw AC displayed a maximum adsorption capacity of 3.5 mg g^−1^ (Kumar et al., 2017). Teak wood sawdust modified using HCl was also shown to enable maximum Cr(VI) adsorption (Jha, 2016).

H_2_SO_4_-activated cashew nut was used for Cr(VI) removal in a column study, which showed the best results at a bed height of 10 cm (Yahya et al., 2020). HCl-activated *M. indica* leaves showed a maximum adsorption capacity of 78.96 mg g^−1^ (Pathania et al., 2020). Vikal et al. (2013) investigated activated *Ziziphus jujuba* leaf powder (local name Bordi), which showed maximum Cr(VI) removal at an adsorbent dose of 15 g within 105 min. Physical activation of the adsorbent was performed at 80°C for 48 h, and FTIR spectroscopy confirmed that the hydroxyl, aliphatic alkane, and O-C stretching of the ether groups were responsible for adsorption. The experimental data were fitted well to both the Langmuir and Freundlich models (Vikal et al., 2013). Sawdust modified with diethylenetriamine also revealed 75% adsorption of Cr(VI) at an optimum adsorbent dosage of 2 g L^−1^ (Elhami and Bahadori, 2015). Chemically (phosphoric acid) activated ECS also showed 87% removal of Cr(VI) at a pH of 3 (Haroon et al., 2020). Spruce sawdust treated with diethylene glycol and sulfuric acid revealed a maximum adsorption capacity of 318.3 mg g^−1^ for Cr(VI) (Politi and Sidiras, 2020). Teakwood sawdust modified with zinc chloride showed an adsorption capacity of 72.46 mg g^−1^ for Cr(VI) (Ramirez et al., 2020). *Leucaena leucocephala* activated with orthophosphoric acid showed a maximum Cr(VI) uptake of 13.85 mg g^−1^ at pH = 4; its PZC was revealed to be 5.42 (Malwade et al., 2016). Chemically activated almond shell (with 40% H_3_PO_4_) was also found to have a high adsorption capacity of 202.34 mg g^−1^ under optimum batch experiments; the BET surface area of the activated almond shells was reported as 1,223.4 m^2^ g^−1^ (Rai et al., 2018). Nut shells activated with ZnCl_2_ were found to have a BET surface area of 2,869 m^2^ g^−1^, and the maximum adsorption was observed at pH = 2; the experimental data were fitted well to the Langmuir model with an adsorption capacity of 46.21 mg g^−1^ (Kumar and Jena, 2017). The adsorption potentials of acid-activated *Juniperus procera* leaves for Pb(II) and Cr(VI) were investigated in a batch experiment, which showed maximum removal efficiencies of 98% and 96% at optimum pH values of 4.6 and 4, respectively. The activated *J. procera* leaves could be reused up to three times, and the data were best fitted to pseudo-first-order kinetics (Ali et al., 2019). Corn cob is an abundant agricultural waste that is mostly burned or discarded. Corn cob activated with a mixture of ZnCl_2_ and NH_4_Cl solutions was explored for Cr(VI) adsorption in a batch experiment, which revealed a BET surface area of 924.9 m^2^ g^−1^; however, the iodine adsorption value of corn cob AC was 1,188 mg g^−1^, and a lower/acidic pH was found to enhance adsorption owing to protonation of the adsorbent surface (Tang et al., 2016).

Sulfuric-acid-activated rice husk showed maximum removal of the adsorbate at a contact time of 120 min and pH of 2. Low desorption results, i.e., 0.1%–9%, revealed strong bonding between the adsorbent and adsorbate as well as the chemisorption nature of the activated adsorbent; the BET surface area was reported to be 58.54 m^2^ g^−1^ (Khan et al., 2016). Sodium-hydroxide-activated carbon prepared from *L. leucocephala* seed pod showed a Langmuir adsorption capacity of 26.94 mg g^−1^ under optimum conditions. The data were best fitted to pseudo-second-order kinetics, indicating chemical adsorption. Furthermore, the desorption efficiency decreased from 63.42% to 47.56% from the first to third cycles as the metal uptake capacity decreased with adsorbent reuse (Yusuff, 2019). *Cicer arietinum* (chickpea) husk was chemically activated using KOH and K_2_CO_3_ and tested for removal of Pb, Cu, and hexavalent Cr; the results showed better porosity formation upon impregnation with KOH (50 wt%), with a BET surface area and total pore volume of 2,082 m^2^ g^−1^ and 1.07 cm^3^ g^−1^, respectively. The maximum adsorption capacities were in the order of Pb(II) > Cr(VI) > Cu(II), i.e., corresponding to 135.8, 59.6, and 56.2 mg g^−1^, and the thermodynamic parameters indicate an endothermic adsorption process (Özsin et al., 2019). Great millet husk was also shown to have an adsorption capacity of 22.21 mg g^−1^ for Cr(VI) removal after chemical modification with sulfuric acid (Prajapati et al., 2022); the metal uptake potential of sulfuric-acid-activated neem bark was tested in a column experiment for hexavalent Cr. When the adsorbent mass was increased from 25 to 175 g at a constant metal ion concentration and flow rate, the breakthrough time was noted to increase from 9.25 to 111.66 h. Activated neem adsorbent has been shown to have excellent effects on a treated mixture of metals (hexavalent Cr, Cu, and Zn) from wastewater; it was revealed that 40% Cr(VI) could be recovered by shaking the exhausted adsorbent with distilled water at 90°C for 2 h, and the data were best fitted to the Yoon–Nelson kinetic model (Maheshwari and Gupta, 2016). *Lantana camara* plant activated with nitric acid displayed a maximum adsorption capacity of 26.25 mg g^−1^ at optimum time, initial concentration, temperature, and pH conditions; the adsorption of Cr(VI) was tested in the presence of interference ions to assess the utility of the activated adsorbent in real industrial effluent applications, and it was observed that the adsorption of Cr(VI) was slightly affected by tenfold excess of co-anions like NO_3_^−^, Cl^−^, and CO_3_^2–^; however, very little effect was noted in the case of SO_4_^2–^ and PO_4_^3–^ (89.0%), while no interference was observed in the case of co-cations like Zn^+2^ (Ravulapalli and Kunta, 2018).

Sulfuric-acid-modified holly sawdust was studied for Cr(VI) treatment in a batch experiment (Siboni et al., 2011); it was observed that the percentage removal of Cr(VI) increased from 34.65% to 99.99% with increasing adsorbent amounts from 2 to 10 g L^–1^. Increasing the initial concentration of the metal ions in the range of 20–100 mg L^–1^ reduced the efficiency from 99.37% to 40.24%. Moreover, the Cr(VI) removal efficiency decreased from 99.67% to 29.78% with increase in pH from 2 to 12. At low or acidic pH, the adsorbent surface acquires positive charges and Cr(VI) generally exists in the form of HCrO_4_^−^. However, the HCrO_4_^−^ changes to CrO_4_^2−^ and Cr_2_O_7_^2−^ at high pH, where the competition between CrO_4_^2−^ and OH^−^ anions also decreases. Equilibrium was attained within 90 min, and the Langmuir model was observed to define the experimental results better than the Freundlich isotherm, with the adsorbent following pseudo-second-order kinetics. The maximum uptake was reported as 18.86 mg g^−1^ at a high pH of 7.0. Two different forms of sawdust, namely, powder sawdust activated with acid (ASDP) and bead-form sawdust with surface modification (SDCCB), were investigated for the treatment of Cr(VI) in a batch study (Begum and Alhaji, 2013). Here, oxidizing agents like H_2_SO_4_, HNO_3_, and H_2_O_2_ were successively used for chemical activation of the sawdust. The highest Cr(VI) uptake capacities for ASDP and SDCCB were 45.5 and 125 mg g^−1^, respectively. The maximum contact times were approximately 120 min for the ASDP and 150 min for the SDCCB. The order of isotherm fitness was Freundlich > Langmuir > Temkin (based on *R* ^2^ values), while pseudo-second-order kinetics was found to best fit the data. It was concluded through the thermodynamic parameters that the adsorption was endothermic, spontaneous, and non-specific chemisorption for both adsorbents (Begum and Alhaji, 2013). The Cr(VI) removal efficiencies of boiled sawdust, formaldehyde-treated sawdust, and sawdust carbon were examined in a batch reactor (Subhan and Pardeep, 2011), which showed that the adsorption process was favored at low a pH of 2.0 when using biomass doses of approximately 4 g L^−1^ and 25°C temperature. The percentage removal efficiency of Cr(VI) was in the following order: sawdust carbon > formaldehyde-treated sawdust > boiled sawdust.

Altun and Pehlivan (2012) investigated the efficiency of chemically modified walnut shell (WNS) (*Juglans regia*) for removal of Cr(VI) from water in a batch experiment; here, approximately 10 g of WNS was chemically modified with 10 g of citric acid per 50 mL of water and heated for 4 h at 120°C, which showed that functional groups like alcohols, carbonyls, carboxyls, and phenols were associated with Cr(VI) adsorption. The equilibrium was attained at 120 min and the optimum pH range was between 2 and 3. The adsorption increased directly with temperature because of more chemical interactions between the adsorbent and adsorbate. The maximum Cr(VI) uptake capacities were 0.596 and 0.154 mmol g^−1^ for CA-WNS and untreated WNS, respectively. The process of adsorption was endothermic and spontaneous (Altun and Pehlivan, 2012). Demirbas et al. (2004) revealed the Cr(VI) removal potential of sulfuric-acid-modified cornelian cherry, apricot, and almond shell from solution. Various environmental parameters like the particle size (0.63–1.60 mm), pH (1–4), and initial Cr(VI) concentration (20–300 mg L^−1^) were optimized in a batch experiment, and the adsorption process was dependent on pH. The equilibrium contact time was 72 h, and the optimum pH was 1. The experimental data were fitted better to second-order kinetics than first-order kinetics in terms of the diffusion model (Demirbas et al., 2004). Attia et al. (2010) conducted a comparative study on sulfuric-acid-modified olive stones and AC to assess their removal potentials for solutions containing various Cr(VI) concentrations (4–50 mg L^−1^) in a batch experiment. The highest removal was reported at the lowest pH of 1.5, and the FTIR spectrum revealed that the negatively charged groups were neutralized to form binding sites for Cr. The adsorption data were suitably fitted to the first-order kinetic and Langmuir models. The AC from olive stones was more efficient than acid-treated commercial AC for Cr(VI) removal (Attia et al., 2010).

*Swietenia mahagoni* shell modified by sulfuric acid and orthophosphoric acid was found to be an excellent adsorbent for the treatment of Cr(VI) in a column. The Cr(VI) adsorption capacities of sulfuric-acid- and orthophosphoric-acid-modified *S. mahagoni* shells from the BDST model were found to be 1,989.4 mg L^−1^ and 2,785.2 mg L^−1^, respectively (Rangabhashiyam et al., 2016). Mane (2010) revealed the potentials of chemically (sulfuric acid) activated tendu (*Diospyros melanoxylon*) leaf refuse (TLR) and commercial activated carbon (CA-CAC) for the treatment of Cr(VI) from solution. The optimum pH was reported as 2, at which point neutralization of negative charges occurred on the adsorbent surface due to excess hydrogen ions, resulting in diffusion or adsorption of the hydrogen chromate ions (HCrO_4_^−^). At pH between 1.0 and 4.0, hydrogen chromate ions are the dominant form of Cr(VI). Another reason for maximum removal at low/acidic pH is the oxidation of Cr_2_O_7_^2–^ to Cr^3+^, which is easily replaced by the positive species owing to its small size. The equilibrium time attained for CA-CAC was 120 min and that for CA-TLR was 30 min. Thus, it was found that CA-TLR is more efficient for Cr(VI) removal than CA-CAC (Mane, 2010). Pandhram and Nimbalkar (2013) explored the efficiency of low-cost activated neem leaves for Cr(VI) removal from industrial effluents; here, the adsorbent was physically activated for 3 h in a furnace at 250°C. In a batch experiment at a pH of 4, the maximum removal observed was 67.5%. At a solution concentration of 30 mg/100 mL, the maximum percentage removal efficiency of Cr(VI) was 98%. At an adsorbent dose of 8 mg/100 mL, the removal efficiency was observed to be maximum at 85% (Pandhram and Nimbalkar, 2013).

The Cr(VI) percentage removal efficiencies of boiled rice husk, formaldehyde-treated rice husk, and rice husk carbon from wastewater were examined in a batch study. The adsorption process was favored at pH = 2 when using an adsorbent dose of 4 g L^−1^ at 25°C. The Cr(VI) removal efficiency was in the order of rice husk carbon > formaldehyde-treated rice husk > boiled rice husk (Subhan and Pardeep, 2011). Ahmed et al. (2012) investigated physically activated rice husk (at 700°C for 1 h) for sequestering Cr from a synthetic aqueous solution using particles of sizes 50 and 100 mesh (150 µm) in batch experiments. The maximum adsorption of 95.2% was attained at an optimum time of 150 min, a Cr(VI) concentration of 20 mg L^−1^, a low pH of 2, and an adsorbent dose of 5 g L^−1^ (Ahmed et al., 2012). *Strychnos potatorum* seeds were modified using sulfuric acid to investigate their efficiency of Cr(VI) removal in both batch and column reactors; here, the maximum Langmuir uptake capacity was reported as 202.7 mg g^−1^, and the column data best fitted the Thomas model (Anbalagan et al., 2016).

Grafted peels of banana displayed 96% Cr(VI) removal at optimum pH and dose of 3 and 4 g L^−1^, respectively (Ali et al., 2016). Pomegranate seeds were also used for Cr(VI) removal (Ghaneian et al., 2017). At an acidic pH of 2, tea husk showed a maximum Cr(VI) removal of 98.5% (Saed, 2016). Sulfuric-acid-activated neem bark was assessed in a column study for Cr(VI) removal, and the result showed increase in breakthrough time from 9.25 to 111.66 h with increase in adsorbent dose (25–175 g). However, the breakthrough time is achieved earlier in multisolutes containing Cu and Zn. The experimental data were explained well by the Yoon–Nelson model. The saturation loading capacity of the column was reported to be 27.5 mg g^-1^, which is very close to the Cr(VI) uptake capacity of 26.95 mg g^-1^ in the batch study (Maheshwari and Gupta, 2016). Stems of the plant *Ocimum tenuiflorum* were activated using sulfuric acid and investigated for Cr(VI) removal in a batch system, where the maximum uptake capacity was found to be 95.9 mg g^-1^. The adsorption mechanism was also evaluated, which showed interparticle diffusion at higher Cr(VI) concentrations and film diffusion at lower Cr(VI) concentrations (Suganya et al., 2017). Sulfuric-acid-modified *S. platensis* with a BET surface area of 765.1 m^2^ g^–1^ was proven to be a good adsorbent and remove up to 55.252% of Cr(VI) even in the presence of sulfate ions; the used adsorbent could be recovered up to 94.48% with sodium hydroxide (Gunasundari and Kumar, 2016).

## 4 Comparisons of adsorbents and chemical modifications for Cr(VI) removal

**FIGURE 7:**
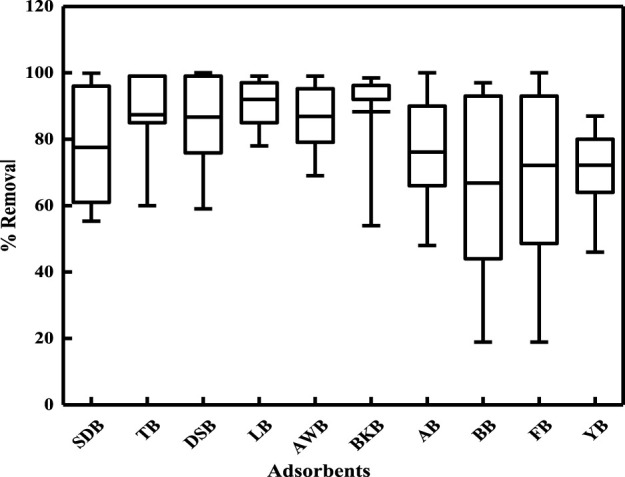
Box-and-whisker plot showing percentage removal of Cr(VI) by various natural biomasses. SDB: sawdust biomass, TB: tea biomass, DSB: dry shell biomass, LB: leaf biomass, AWB: agricultural waste biomass, BKB: bark biomass, AB: algal biomass, BB: bacterial biomass, FB: fungal biomass, YB: yeast biomass.

Previous reports in literature have revealed the following order for average Cr(VI) removal (Figure 7) when using different biomasses without any modifications or activation: leaves > bark > agricultural waste > dry shells > tea = fungi > yeast > algae > sawdust > bacteria. The adsorption data of different types of leaves are best fitted to both the Langmuir and Freundlich isotherm models, reflecting single-layer physisorption as well as multilayer chemisorption. Leaf modifications do not increase the maximum adsorption efficiency, so it is economical to use leaves that are available in bulk refuse without prior treatment with chemicals in various industries, especially in developing countries. Studies also show that adsorption depends on the solution pH, functional groups on the adsorbent surface, particle size of the adsorbent, and metal-ion concentration.

**FIGURE 8:**
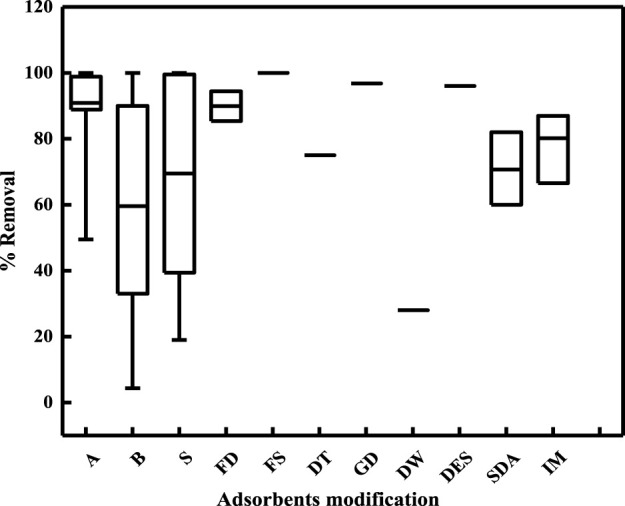
Box-and-whisker plot showing percentage removal of Cr(VI) by modifications of natural adsorbents. A: acid, B: base, S: salt, FD: formaldehyde, FS: formaldehyde with sulfuric acid, DT: diethylenetriamine, GD: glutaraldehyde, DW: distilled water, DES: diethyl ether and sulfuric acid, SDA: sodium alginate, IM: immobilized.

The physical and/or chemical modifications enhanced Cr(VI) removal with almost all natural lignocellulosic (organic) adsorbents. The major mechanisms driving metal-ion adsorption are electrostatic interactions, surface complexation, and ion exchange. Modification of the adsorbents with acids produced the best results for Cr(VI) removal, followed by modifications with salts and bases (Figure 8). Modification with an acid results in positive charges on the adsorbent surface (Abegunde et al., 2020). Modification of agricultural or organic materials with acids through the wet oxidation process causes dissolution of the constituents or removal of mineral impurities along with an increase in oxygen content and a decrease in pore tortuosity. Modification of organic adsorbents with various acids increases the reactivity of the functional groups and different surface properties of the adsorbents, such as the surface area and porosity, thereby increasing adsorption. Acid modification can also increase the hydrolysis of cellulose, which results in a more reactive adsorbent compared to the untreated adsorbent (Liu et al., 2020). Modification of adsorbents with acids also reduces the mineral content of the modified adsorbent, which in turn results in increased percentage removal of Cr(VI) from water because of the decrease in competition between the active sites of the adsorbent and cations. Sulfuric acid is the most widely used substance for modification and increases the removal efficiency by 80%–100% in all the reviewed adsorbents. This is because of the presence of -SO_3_ groups on the adsorbent that increases the percentage removal of Cr(VI) through covalent bonding along with increases in the surface area and porosity of the adsorbent (Goswami and Phukan, 2017). Phosphoric-acid- and tartaric-acid-based modifications are also reported in literature, and these enhance the porosities and surface areas of natural adsorbents. Modifications of adsorbents with sulfuric acid, phosphoric acid, and nitric acid increase both the surface areas and surface functional groups (Lesaoana et al., 2019). Modification with phosphoric acid has been reported to form different phosphorus groups on the adsorbent surface (PO and POOH), which then react with Cr(VI) and result in the formation of metal complexes (Kainth et al., 2024). However, in the case of modification using nitric acid, there is enhancement of the nitrate groups along with increased pore volume on the adsorbent surface, which results in more adsorption of Cr(VI). Sodium hydroxide as a modification source for biomass showed poor results because of fewer positive charges, as in the case of acid modification. The optimum pH was found to be 2 in most of the studies with both raw and modified adsorbents. In the case of treated biomass, the Langmuir model was found to be the best fit most often, indicating the availability of more surface sites for Cr(VI) removal. It is important to note that the different chemical modifications summarized in Figure 8 are generally applicable for all lignocellulosic (organic) biomasses because of their similar structural compositions; further, the best modification (chemical) may differ based on specific biomass properties along with the experimental conditions noted in the various studies.

In the box-and-whisker plots shown in Figures 7 and 8, the rectangles (boxes) represent the interquartile range (IQR; range between the 25th and 75th percentiles) while the lines (whiskers) represent the spread of the data, which extend to the minimum and maximum values (excluding outliers). The central line within each box indicates the median value.

## 5 Future directions

In most of the reported works, researchers have focused on batch experimentation; however, it is important to work on column designs using natural adsorbents for field applications. Hence, further exploration is needed for the development of novel and efficient natural adsorbents from natural materials, such as functionalized adsorbents, nanocomposites, engineered biochars, and hybrid materials. More studies are needed on the treatment of real industrial wastewater and effluents containing multiple pollutants as there is less available work on this area given that most of the literature is on synthetic solutions of single adsorbates. In most cases, the Freundlich and Langmuir isotherms were used for the analyses while there are very few reported cases of using other models like the Elovich, D-R, and Temkin isotherms; this area also needs to be explored further for better understanding of the nature of adsorption processes. Exploration of the microscopic mechanisms of adsorption is also needed using advanced techniques like X-ray photoelectron spectroscopy and density functional theory simulations. The development of cost-effective methods to produce AC from locally available biomasses is the most desired need for field applications. Hence, more research efforts are required for optimizing methods for economical development of AC from various available waste biomasses. Mixtures or compositions with varying ratios of the activated and non-activated adsorbents should also be evaluated for pollutant removal. Low regeneration has been observed with most of the adsorbents; therefore, further desorption/regeneration processes should be studied using different variables like pH, temperature, rotation speed, chemicals, etc. for renewal of exhausted adsorbents. Another area for future development is the exploration of hybrid or synergetic treatment approaches in which adsorption is combined with other technologies like biological treatments, membranes, electrochemical reduction, photocatalysis, and advanced oxidation processes. There is also a need to carry out lifecycle assessments of the Cr adsorption processes. Lastly, we note the importance of conducting cost-benefit analyses to better understand the practical applications of the adsorbents.

## 6 Conclusion

The bulk of organic biomass is often treated as waste and refuse all around the world. The present review on these waste biomasses reveals their effectiveness as low-cost adsorbents for the removal of Cr(VI) from wastewater. Our survey indicates the following order of utility of various biomasses for Cr(VI) removal: leaves > bark > agricultural waste > dry shells > tea = fungi > yeast > algae > sawdust > bacteria. Among the various types of bulk biomasses available, leaves have been proven to be the most efficient and economical sources for Cr(VI) removal that do not require any prior treatment. The optimum pH for the treatment was found to be 2 for both raw and modified adsorbents. The major mechanisms driving metal ion adsorption are electrostatic interactions, surface complexation, and ion exchange. Both the Langmuir and Freundlich isotherms show best fits to the adsorption data in most cases, which is indicative of both physisorption and chemisorption mechanisms. Previous literature also indicates that the adsorption depends on the solution pH, surface functional groups on the adsorbent, particle size of the adsorbent, and metal ion concentration in the solution. Lastly, we recommend that the commercial application of these adsorbents be enforced and a standardized practice be established for the treatment of wastewater at the industrial level.
